# Synthesis of high-entropy alloy nanoparticles on supports by the fast moving bed pyrolysis

**DOI:** 10.1038/s41467-020-15934-1

**Published:** 2020-04-24

**Authors:** Shaojie Gao, Shaoyun Hao, Zhennan Huang, Yifei Yuan, Song Han, Lecheng Lei, Xingwang Zhang, Reza Shahbazian-Yassar, Jun Lu

**Affiliations:** 1 Key Laboratory of Biomass Chemical Engineering of Ministry of Education, College of Chemical and Biological Engineering, Zhejiang University, 310027 Hangzhou, Zhejiang Province China; 2 Department of Mechanical and Industrial Engineering, University of Illinois at Chicago, Chicago, IL 60607 USA; 3 Chemical Sciences and Engineering Division, Argonne National Laboratory, 9700 S. Cass Avenue, Lemont, IL 60439 USA; 4 School of Environment and Safety Engineering, Jiangsu University, 212013 Zhenjiang, Jiangsu Province China; 5 Institute of Zhejiang University-Quzhou, 78 Jiuhua Boulevard North, 324000 Quzhou, China

**Keywords:** Electrocatalysis, Synthesis and processing

## Abstract

High-entropy alloy nanoparticles (HEA-NPs) are important class of materials with significant technological potential. However, the strategies for synthesizing uniformly dispersed HEA-NPs on granular supports such as carbon materials, γ-Al_2_O_3_, and zeolite, which is vital to their practical applications, are largely unexplored. Herein, we present a fast moving bed pyrolysis strategy to immobilize HEA-NPs on granular supports with a narrow size distribution of 2 nm up to denary (MnCoNiCuRhPdSnIrPtAu) HEA-NPs at 923 K. Fast moving bed pyrolysis strategy ensures the mixed metal precursors rapidly and simultaneously pyrolyzed at high temperatures, resulting in nuclei with a small size. The representative quinary (FeCoPdIrPt) HEA-NPs exhibit high stability (150 h) toward hydrogen evolution reaction with high mass activity, which is 26 times higher than the commercial Pt/C at an overpotential of 100 mV. Our strategy provides an improved methodology for synthesizing HEA-NPs on various supports.

## Abstract (web summary)

The large-scale application of extremely small, high-entropy alloy nanoparticles is limited by the phase separation and immobilization. Here, the authors develop a general method of fast-moving bed pyrolysis, uniformly dispersing high-entropy alloy nanoparticles on various granular supports.

## Introduction

High-entropy alloys, containing five or even more metals, have attracted significant attentions^1–5^, because of their unique chemical and physical complexity, which endow alloys with tunable features and properties such as thermal stability, superior corrosion resistance, high hardness, exceptional ductility, and superparamagnetism^6–10^. A wide range of researches have revealed that the nanoparticles loading on the supports could significantly improve specific surface area and surface energy compared with the bulk materials or the nanoparticles themselves^11,12^. In addition, in industrial applications, the nanoparticles need to be dispersedly immobilized on granular supports, such as Al_2_O_3_, zeolite, and carbon materials etc., which can make the catalysts feature with tunable size, shape, and stability, enhancing the catalytic performance^13–15^. Therefore, the supported high-entropy alloy nanoparticles (HEA-NPs) can be promisingly applied in catalysis, chemical sensing, biology, and energy conversion^16–21^. However, the common extreme high temperature related approaches such as arc melting^16^ and laser cladding^22^ are not suitable for supporting HEA-NPs due to the rapid growth and aggregation of nanoparticles^23^. Although multimetallic nanoparticles can be produced at relatively mild conditions, including magnetron sputtering^24^, the mixed metal oxides inking^25^, electrodeposition^26^, and polymer lithography methods^27,28^, which are difficult for uniformly immobilizing HEA-NPs on granular supports. Notably, Yao et al. applied carbothermal shock method successfully supported 8 elemental HEA-NPs on conductive activated carbon fiber through flash heating and cooling strategy^29^, but it is very challenging for this joule heating method to immobilize HEA-NPs on granular supports (independent of their electrical conductivity) such as active carbon, alumina oxide, and zeolite. Exploring the efficient method for immobilizing HEA-NPs on these granular supports is obviously significant for facilitating the industrial application of HEA-NPs and providing facile platform for designing catalysts and understanding support-metal interaction effect.

Presently, wet impregnation followed by reductive pyrolysis in the programmed temperature heating way, which is always conducted in the fixed bed pyrolysis (FBP) reactor, is the most popular method for preparation of supported nanoparticles on granular supports^13,14^. However, in the conventional FBP process, the metal ions are sequentially reduced due to the different starting reductive temperatures of precursors, which are mainly resulted from the different chemical reductive potentials^30,31^. According to the classical LaMer mechanism of the formation of nanocrystals, the higher supersaturation of monomers can induce a burst nucleation and the smaller nuclei formation^32–35^. The theoretical and experimental results of nanoalloying have shown that decreasing critical size of nuclei could reduce the excess free energy cost for formation of nanoalloys avoiding phase separation^36–41^.

Here we show a general and facile fast moving bed pyrolysis (FMBP) strategy following wet impregnation for the preparation of ultrasmall and highly dispersed HEA-NPs coming up to 10 immiscible elements (Mn, Co, Ni, Cu, Rh, Pd, Sn, Ir, Pt, and Au) via the pyrolysis of the mixed metal chlorides precursors loading on various granular supports such as carbon support (carbon black and graphene oxide), γ-Al_2_O_3_, and zeolite (Permutit). In the FMBP process, the formation of HEA-NPs is thermodynamically favored due to the low free energy of the formation of nuclei, which results from the fast pyrolysis of precursors at high temperatures. The representative quinary (FeCoPdIrPt) HEA-NPs possess the high activity and exceptional stability toward hydrogen evolution in water splitting.

## Results

### The FMBP strategy for HEA-NPs

**Fig. 1:**
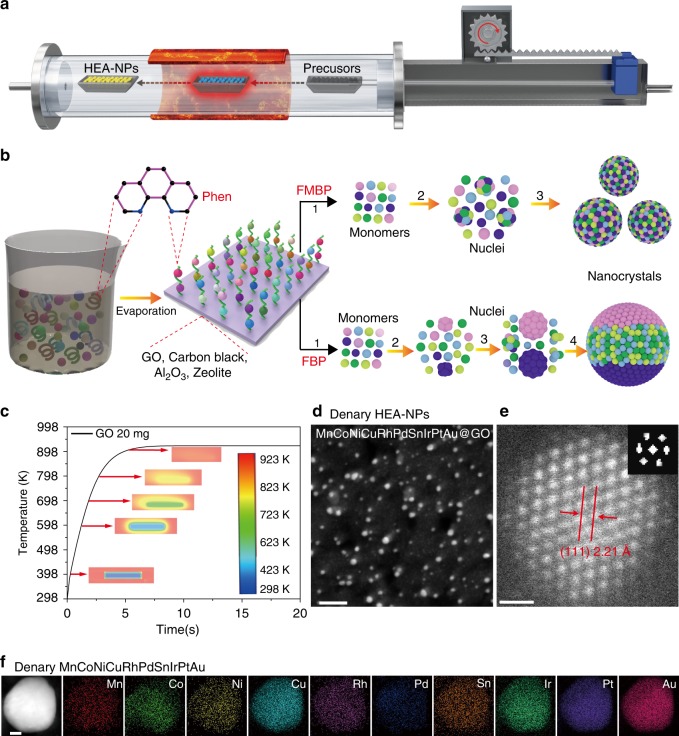
The FMBP strategy for synthesis of HEA-NPs. **a** Schematic diagram of the FMBP experimental setup for synthesis of HEA-NPs. **b** Schematic diagrams for synthesis of homogeneous and phase-separated HEA-NPs by FMBP and FBP strategies, respectively. **c** The simulation of the time required for precursors/GO (20 mg, 3 wt%) to reach 923 K in the FMBP process. Center: the metal precursors/GO in the quartz boat. **d** HAADF-STEM images for the denary (MnCoNiCuRhPdSnIrPtAu) HEA-NPs highly dispersed on GO synthesized by the FMBP strategy (The loading of HEA-NPs on GO was 3 wt%). **e** The HR-STEM image for the denary (MnCoNiCuRhPdSnIrPtAu) HEA-NPs (inset, the Fourier transform analysis for denary (MnCoNiCuRhPdSnIrPtAu) HEA-NPs indicated that the denary HEA-NPs featured with an fcc crystal framework). **f** Elemental maps for denary (MnCoNiCuRhPdSnIrPtAu) HEA-NPs (The loading of HEA-NPs on GO was 10 wt%). The elements in HEA-NPs have the equal atomic ratio. Scale bar **d**: 10 nm, **e**: 0.5 nm, and **f**: 10 nm.

Our FMBP strategy allows the metal precursors to rapidly reach 923 K within 5 s (a propulsion speed of 20 cm s^−1^) with the temperature in the heating zone only dropping to 920 K (Fig. 1a and Supplementary Movie 1). The FMBP strategy ensures the simultaneous pyrolysis of the mixed metal precursors (Supplementary Figs. 1 and 2) due to rapidly reaching the high temperature (above the pyrolysis temperature of all precursors), resulting in the high supersaturation of monomers, in turn forming smaller nuclei clusters to form HEA-NPs without phase separation (Fig. 1b). In contrast, the FBP strategy can only synthesize phase-separated alloy, because each metal precursor would be reduced in sequence for their various reduction potentials during temperature programming (20 K min^−1^) (Fig. 1b). Moreover, we simulate the heat transfer of 20 mg of precursors/graphene oxide (GO) placed in a quartz boat (the commercial ANSYS FLUENT software). The results showed that GO could reach 923 K within 5 s, which was consistent with the time of the actual experimental operation (Fig. 1c, Supplementary Movie 2).

### Crystal structure and composition characterization

As-proof-concept, the denary (MnCoNiCuRhPdSnIrPtAu) HEA-NPs were synthesized by the FMBP strategy. Firstly, the mixed denary metal chloride salts precursors coordinated with 1,10-Phenanthroline (Phen) were loaded on GO by the wet impregnation method, then, followed by the reductive pyrolysis to obtain HEA-NPs at 923 K through the FMBP strategy (details in experimental section). It was seen that the denary (MnCoNiCuRhPdSnIrPtAu) HEA-NPs were uniformly dispersed on GO with an ultrasmall size ~2 nm (Fig. 1d). The high-resolution scanning transmission electron microscope (HR-STEM) revealed that the crystalline structure of denary HEA-NPs was face-centered cubic (fcc) (Fig. 1e). Additionally, the elemental maps indicated that 10 elements were uniformly distributed in the nanoparticles, suggesting that the denary HEA-NPs were atomically mixing without phase separation (Fig. 1f). It should be pointed out that the HEA-NPs with a low loading of HEA-NPs on GO (3 wt%) were too small to obtain clear mapping images in high resolution with energy-dispersive X-ray (EDX) spectroscopy, so we used a high loading of HEA-NPs on GO (10 wt%), as shown in Fig. 1f.

**Fig. 2:**
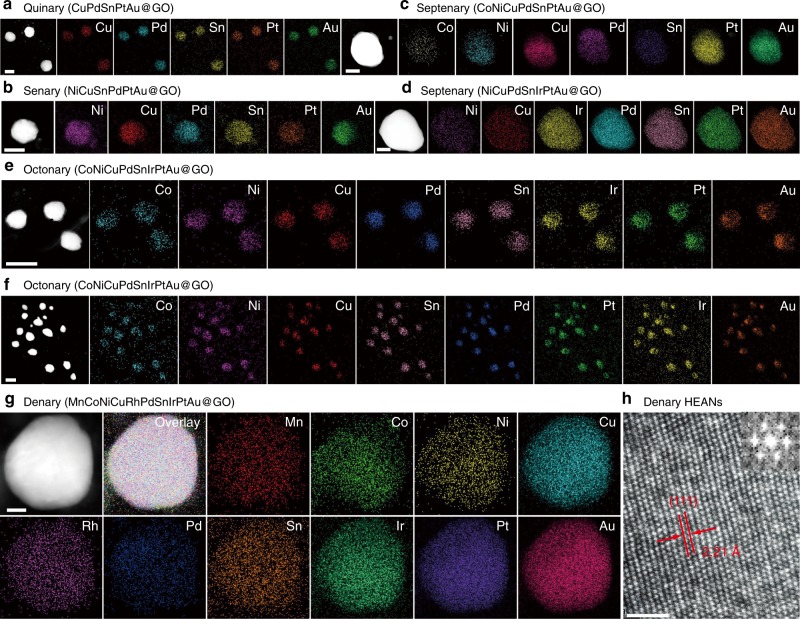
HAADF and elemental maps for HEA-NPs synthesized by FMBP. HAADF and elemental maps for the quinary (CuPdSnPtAu) (**a**), senary (NiCuPdSnAuPt) (**b**), septenary (CoNiCuPdSnPtAu) (**c**), septenary (NiCuPdSnIrPtAu) (**d**), octonary (CoNiCuPdSnIrPtAu) (**e**) and (**f**), and denary (MnCoNiCuRhPdSnIrPtAu) alloy (**g**), and supported on GO. **h** The high-resolution TEM image for the denary (MnCoNiCuRhPdSnIrPtAu@GO) HEA-NPs. The inset shows the Fourier transform analysis for denary HEA-NPs indicating that the denary HEA-NPs exhibited an fcc crystal framework. The loading of HEA-NPs on GO was 10 wt%. The elements in HEA-NPs have the equal atomic ratio. Scale bar (**a**–**c**, **e**, **f**): 50 nm, **d**: 20 nm, **g**: 10 nm, and **h**: 1 nm.

Our FMBP strategy can facilely synthesize a wide range of HEA-NPs, including the quinary (e.g., CuPdSnPtAu), senary (e.g., NiCuPdSnPtAu), septenary (e.g., NiCuPdSnIrPtAu), and octonary (e.g., CoNiCuPdSnIrPtAu) alloy at 923 K (Fig. 2), illustrating the reliability and generality of the FMBP strategy for synthesis of HEA-NPs. The elemental maps clearly revealed that each element in these alloys supported on GO was uniformly mixed without phase separation (Fig. 2a–g). Besides that, the corresponding elemental maps for the atomic scale HAADF-STEM image of CuSnPdAuPt HEA-NPs further revealed these elements were uniformly distributed in these HEA-NPs (Supplementary Fig. 3). Additionally, high-resolution high-angle annular dark-field imaging in scan transmission electron microscopy (HAADF-STEM) showed that the lattice fringe spacing in the denary nanoparticle (50 nm) was 2.21 Å, which was well identical with that in the denary nanoparticle with the size of 2 nm (Fig. 1e), revealing that the nanoparticles in the denary high-entropy alloy forming homogeneous alloys. Simultaneously, the formation of quinary (CuPdSnPtAu) and octonary (CoNiCuPdSnIrPtAu) HEA-NPs were also confirmed by HAADF-STEM (Supplementary Figs. 3 and 4). Additionally, in order to prove that the synthesized alloy nanoparticles are atomically mixed without phase segregation, the representative quinary and denary HEA-NPs were characterized with powder X-ray diffraction (XRD), which revealed that the homogeneous HEA-NPs by the FMBP strategy were successfully synthesized (Supplementary Figs. 5 and 6).

The composition ratio of each element in these HEA-NPs was analyzed through EDX (Supplementary Figs. 7–13, showing that the atomic ratio of each element was very close to the expected ratio. The composition of individual particles (FeCoPdPtIr HEA-NPs) was further analyzed to prove each element in HEA-NPs is uniformly distributed. The atomic ratio of each element was close to the average compositions (Supplementary Fig. 14). The atomic ratio error in these alloys is below 15%, which may be due to the possible evaporation of metal precursors and the analysis error. In order to further prove that the proportion of each element is equal to the desired ratio of metals, we used inductively coupled plasma optical emission spectroscopy (ICP-OES) to analyze the composition of HEA-NPs. The results showed that the element ratio of the quinary, octonary, and denary HEA-NPs was very close to the results obtained by the EDX (Supplementary Tables 1–3). Furthermore, the ternary, octonary, and denary HEA-NPs were respectively characterized with X-ray photoelectron spectroscopy (XPS) (Supplementary Figs. 15–19), confirming the homogenous HEA-NPs. Thus, this indicates the generality of our method to achieve various mixing compositions in HEA-NPs on different substrates.

### Immobilizing HEA-NPs on various supports

**Fig. 3:**
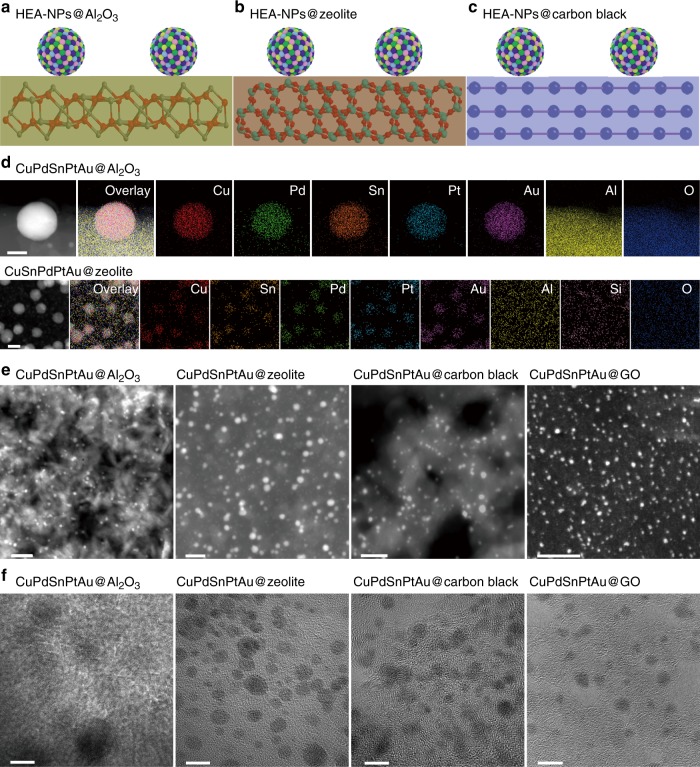
Supporting HEA-NPs on various supports. The schematic diagrams for HEA-NPs dispersed on γ-Al_2_O_3_ (**a**), zeolite (**b**), and carbon black (**c**). **d** The elemental maps for quinary (CuPdSnPtAu) supported on Al_2_O_3_ and zeolite, (The loading of HEA-NPs on γ-Al_2_O_3_ and zeolite was 10 wt%). **e** STEM images revealed that the HEA-NPs synthesized by FMBP strategy were highly dispersed on γ-Al_2_O_3_, zeolite, carbon black, and GO. **f** HR-TEM images for HEA-NPs supported on γ-Al_2_O_3_, zeolite, carbon black, and GO synthesized by FMBP strategy (The loading of HEA-NPs on supports was 3 wt%). The elements in HEA-NPs have the equal atomic ratio. Scale bar **d**: 10 nm, **e**: 20 nm, **f**: 5 nm.

Our FMBP strategy can be readily extended to immobilize HEA-NPs on various granular supports including γ-Al_2_O_3_, zeolite, and carbon black (Fig. 3), taking the quinary (CuPdSnPtAu) HEA-NPs as an example. It should be pointed out that when γ-Al_2_O_3_ or zeolite was used as supports, the reduction of metal precursors was achieved with H_2_ (100 sccm) under 923 K. As shown in Fig. 3d, the elemental maps clearly revealed that elements in the quinary (CuPdSnPtAu) HEA-NPs on γ-Al_2_O_3_ and zeolite were also uniformly mixed without phase separation, suggesting formation of homogenous alloys, in agreement with results of Fig. 2 (GO as supports). These results indicated that our FMBP method is general to immobilize HEA-NPs on various supports. Moreover, the HEA-NPs synthesized by our FMBP method exhibited a uniform and small particle size distribution (~5 nm) when the loading of alloys was 3 wt% (Fig. 3e and Supplementary Figs. 20 and 21). For example, the sizes of these particles from 1.5 to 2.5 nm occupy 73.8% in the size distribution for the denary (MnCoNiCuRhPdSnIrPtAu@GO) HEA-NPs (Supplementary Fig. 21). These HEA-NPs are mostly smaller than 5 nm in diameter and uniformly dispersed on carbon supports (GO and carbon black), γ-Al_2_O_3_, and zeolite. It should be noted that the HR-TEM pictures of HEA-NPs supported on GO, carbon black, γ-Al_2_O_3_, and zeolite all showed clear crystal lattices (Fig. 3f, Supplementary Figs. 7–13), suggesting HEA-NPs were highly crystalline.

### Effect of temperature on alloy formation

**Fig. 4:**
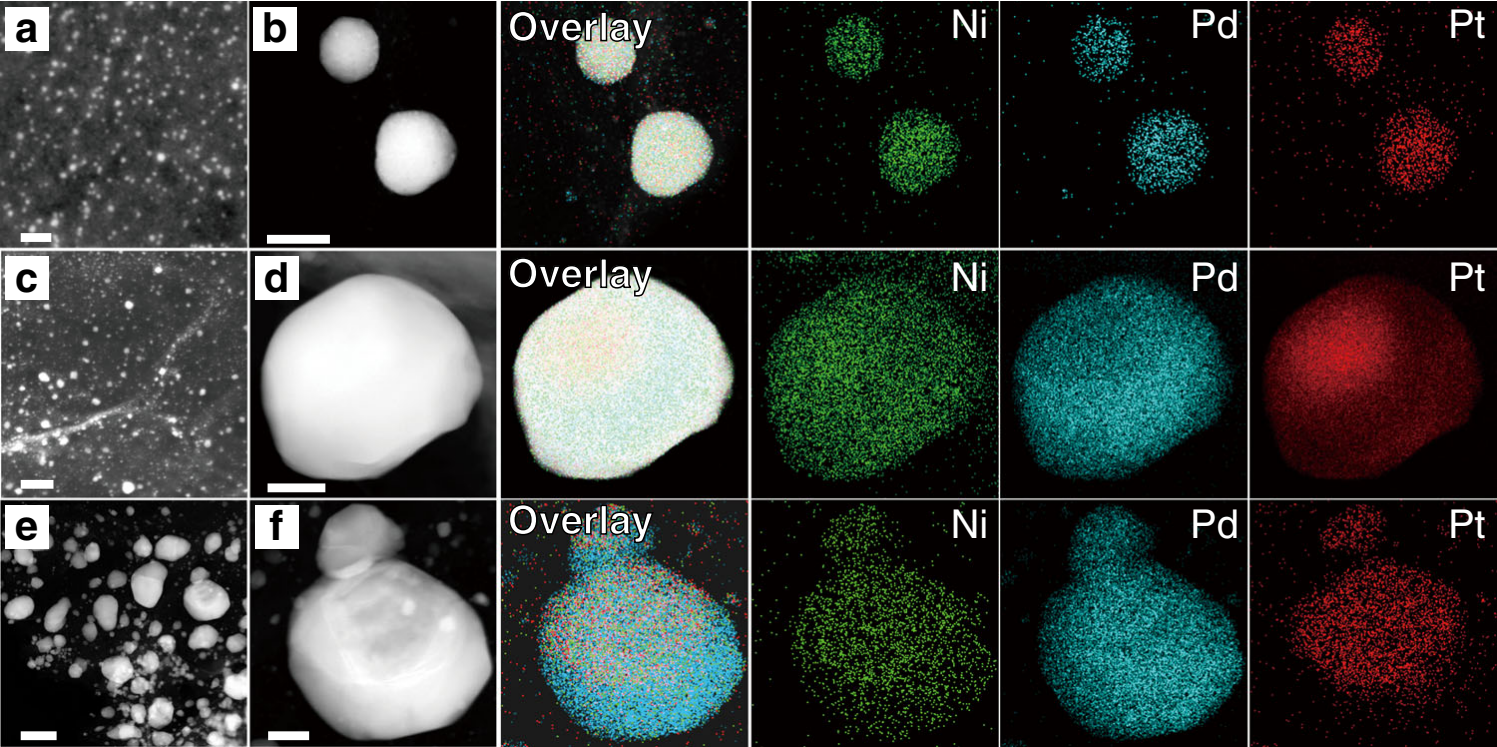
Synthesis of NiPdPt by different methods. **a** The STEM image of NiPdPt obtained by FMBP (923 K). **b** The elemental maps of NiPdPt obtained by FMBP (923 K). **c** The STEM image of NiPdPt obtained by SMBP (923 K). **d** The elemental maps of NiPdPt obtained by SMBP. **e** The STEM image of NiPdPt obtained by FBP. **f** The elemental maps of NiPdPt obtained by FBP. The loading of HEA-NPs on GO was 3 wt% for **a**, **c**, **e**. The loading of HEA-NPs on GO was 10 wt% for the NiPdPt alloy for elemental maps. Scale bar **a**: 10 nm, (**b**–**d**, **f**): 50 nm, **e**: 200 nm.

We carried out control experiments to verify the assumption that the strategy of pyrolysis reduction of mixed metal precursors governed the formation of alloys (Fig. 4). We took the ternary (NiPdPt@GO) alloy as an example to investigate the influence of different pyrolysis strategies on alloy formation, because more elements will make the characterization much more complicated and challenging. In the FMBP process, the NiPdPt alloy was formed without phase segregation (Fig. 4b). In contrast, as shown in Fig. 4d, when the propulsion speed was decreased to 1 cm s^−1^ (20 s for precursors to reach the heating zone), namely the slow moving bed pyrolysis (SMBP), the phase-separated NiPdPt nanoparticle was clearly formed, where Pt and Pd were not uniformly in the NiPdPt nanoparticles. More obviously, Pd only existed in the core of NiPdPt nanoparticles obtained by the conventional FBP strategy (923 K) with a heating rate of 20 K min^−1^ (~31 min from 298 K to 923 K), indicating a completely phase segregation (Fig. 4f, Supplementary Fig. 22). Besides that, the pyrolysis strategy also changed and affected the particle size of the synthesized alloy (Fig. 4a, c, e). These controls clearly illustrated the key role of the pyrolysis strategies of the mixed metal precursors for formation of HEA-NPs.

### Mechanism of formation of HEA-NPs

**Fig. 5:**
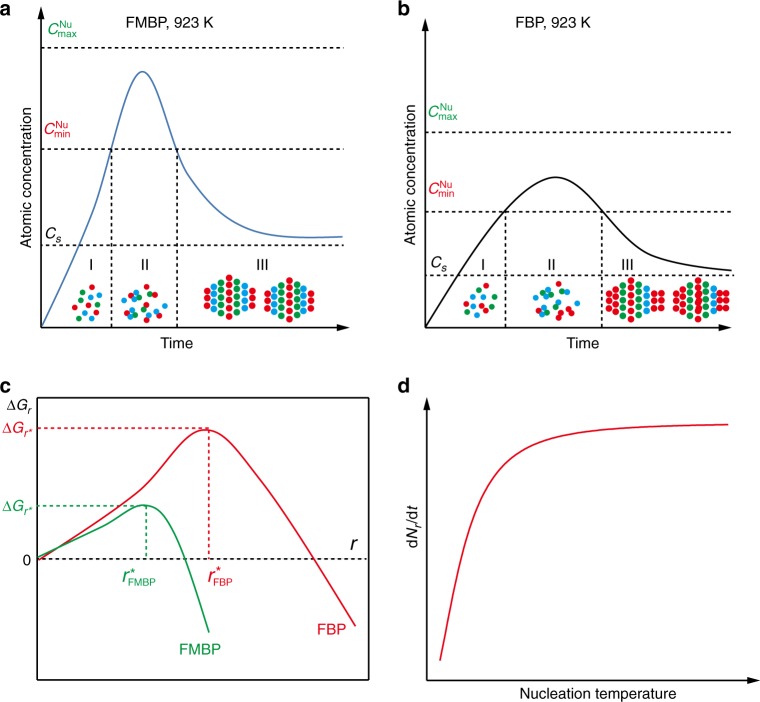
Mechanism of formation of nanocrystals by FMBP and FBP. **a** The diagrams of formation of monomers (I), nucleation (II), and growth of nanocrystal (III) versus the reaction time by FMBP. **b** The diagrams of formation of monomers (I), nucleation (II), and growth of nanocrystal (III) phases versus the reaction time by FBP. **c** The corresponding overall free energy change ((∆*G*_*r*_) versus the nucleus size (*r*) via FMBP, and FBP strategies. **d** The effect of nucleation temperature on the nucleation rate (d*N*_*r*_/d*t*).

The formation of nanocrystals is generally divided into three steps according to the LaMer’s nucleation mechanism (Fig. 5a, b)^32,39,42^:

$${\mathrm{Precursors}} \to {\mathrm{Monomers}} \leftrightarrow {\mathrm{Nuclei}} \to {\mathrm{Nanocrystal}}$$

The correlation of free energy of nuclei with HEA-NPs is significant for nuclei, because different energy can lead to forming alloys or phase-separated nanocrystal. The overall free energy change versus the nucleus size during the formation of nuclei (Fig. 5c and Supplementary Fig. 23) as follows^33,34,39^:

1 $${\mathrm{\Delta }}G_r = 4{\uppi}r^2\gamma + \frac{4}{3}\pi r^3{\mathrm{\Delta }}G_V$$

2 $${\mathrm{\Delta }}G_V = - \frac{{RTlnS}}{{V_m}}$$

3 $${\mathrm{\Delta }}G_r = 4{\uppi}r^2\gamma + \frac{4}{3}\pi r^3{\mathrm{\Delta }}G_V = 4{\uppi}r^2\gamma - \frac{4}{3}\pi r^3\frac{{RTlnS}}{{V_m}}$$

Where *r* is the nucleation radius, *γ* is surface free energy in per unit area, *R* is the ideal gas constant, ∆*G*_*V*_ is the free energy gap for per unit volume of metal solids and solute particles, *T* is the temperature for nucleation, *V*_*m*_ is solid molar volume, *S* is the ratio of supersaturation concentration to equilibrium concentration, and ∆*G*_*r*_ is excess free energy, which is related with the surface energy (4*πr*^2^*γ*) and volume free energy (4/3)*πr*^3^∆*G*_*V*_.

As shown in Fig. 5c, when the radius of nuclei is smaller than the critical value, the nuclei is easily decomposed, and the new nuclei forms resulting from the aggregation of monomers or the small nuclei. When the nucleus size is larger than the critical radius (r*), the nucleation system will decrease its free energy by the growth of bigger clusters^39^. The critical radius r* and the corresponding critical excess free energy ∆*G*_r*_ can be described as the followings by solving the equation d*G*_*r*_/d*r* = 0:

4 $$r^\ast = \frac{{2\gamma V_m}}{{RTlnS}}$$

5 $${\mathrm{\Delta }}G_{r^ \ast } = 4{\uppi}r^{ \ast 2}\gamma - \frac{4}{3}{\uppi}r^{ \ast 3}\frac{{RTlnS}}{{V_m}}$$

**Fig. 6:**
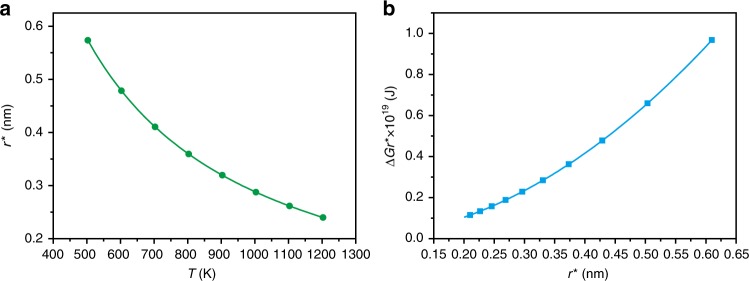
The quantitative analysis of nucleation process. **a** The critical radius of nuclei (r*) as functions of nucleation temperature (*T*). **b** The critical excess free energy (∆*G*_r*_) as functions of critical radius (r*).

Therefore, we can get the quantitative relation between the temperature and critical radius of nuclei (r*) according to Eq. 4 (Fig. 6a). Then, the quantitative relation between the critical excess free energy (∆*G*_r*_) and the critical radius (r*) can be further calculated according to Eq. 5 (Fig. 6b and Supplementary Tables 4 and 5). Obviously, the reaction temperature has a strong effect on the critical radius of nuclei (r*) and the critical excess free energy (∆*G*_r*_), for example, at 923 K, r*, and ∆*G*_r*_ were 0.313 nm and 2.54 × 10^−20^ J, respectively (Supplementary Table 6). In contrast, at 673 K, r*, and ∆*G*_r*_ were 0.442 nm and 5.08 × 10^−20^ J, respectively.

In our FMBP, the mixed metal precursors could rapidly reach the high temperature (e.g. 923 K). The time for the supports such as GO, zeolite, and γ-Al_2_O_3_ supporting precursors to reach 923 K in the FMBP process were simulated. The results revealed that these supports could reach 923 K within 5 s, showing almost no difference with the practical operation (Supplementary Fig. 24 and Movies 1 and 2). It should be noted that all metal precursors with different properties could be efficiently decomposed under 923 K (Supplementary Tables 4–8). Therefore, in the FMBP process (Fig. 5a, c), the formation of HEA-NPs was thermodynamically favored due to the low critical excess free energy (∆*G*_r*_)^39–42^. The nuclei of alloys with the small size will be much favorable due to the fact that these monomers only need little energy for nucleation^43,44^. In contrast, in FBP (Fig. 5b) or SMBP, the formation of alloys slowly going through the low temperature (e.g. 673 K) with the slow heating way would result in the larger radius of nuclei, and the phase separation would occur due to the high ∆*G*_r*_ (Fig. 4c–f). The total number of the nuclei clusters obeys the Boltzmann distribution, and the increase of nuclei radius will decrease the number of nuclei^35,39,40^. The higher nucleation temperature is beneficial to the nucleation rate (Fig. 5d, Supplementary Fig. 25, and Supplementary Note 1). Therefore, in our FMBP, the homogenous HEA-NPs with small size (2 nm) were easily formed due to the smaller nuclei radius and the fast nucleation rate.

We investigated the pyrolysis reduction temperatures of metal precursors on the formation of alloys in the FMBP process. Also taking the ternary NiPdPt alloy as example, it was obviously seen that NiPdPt alloy cannot be obtained under the pyrolysis temperature of 673 K. The Ni element was scattered on the GO support, and the bimetallic PdPt alloy can be formed due to their similar chemical reduction potentials (Supplementary Fig. 26). When the reduction pyrolysis temperature increased to 923 K and 1173 K, the NiPdPt alloy was successfully synthesized with uniformly mixing of Ni, Pt, and Pd (Fig. 4b and Supplementary Fig. 26). However, the NiPdPt alloy synthesized with FMBP under 1173 K produced larger and aggregated HEA-NPs particles (Supplementary Fig. 27). Additionally, according to the calculated free energy and Boltzmann distribution, the rate of the transformation of metal precursors to nuclei clusters (*d*_*N*_/*d*_*t*_) was close to 1 at 923 K, when *S* (the ratio of supersaturation concentration to equilibrium concentration) was in range of 5–20 (Supplementary Fig. 25). The nucleation rate directly determined the size and number of nuclei generated in the supersaturation of monomers. The faster the pyrolysis rate of precursors, the supersaturation of monomer will be higher. Thus, the larger the number of the nuclei clusters, and the smaller the nanocrystals generated. Overall therefore, the reasonable reduction pyrolysis temperature of 923 K is important for our FMBP strategy to get the HEA-NPs with small size.

Actually, the annealing time for synthesizing HEA-NPs was also investigated in detail, including 30 min, 120 min, and 180 min. Different annealing time will affect the formation of high-entropy alloys. When the annealing time was set at 30 min, the CuSnPdPtAu HEA-NPs would be wrapped by carbon (Supplementary Fig. 28), which cannot be sufficiently decomposed. Moreover, as can be seen from Supplementary Fig. 28, when the annealing time was set at 180 min, the size of CuSnPdPtAu HEA-NPs would significantly increase. Thus, the annealing time of 120 min was chosen as the suitable time for synthesizing the ultrasamll HEA-NPs. Besides that, the selective reaction time was also based on the diffusion of reactants and draining of gases produced from precursors.

### HER tests

**Fig. 7:**
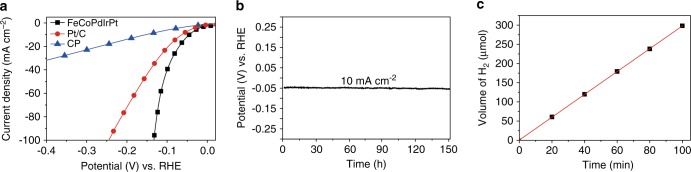
Electrochemical HER performance of samples. **a** The activities toward HER of the prepared FeCoPdPtIr@GO and Pt/C, and pure CP eOur FMBP strategy allowslectrodes. Linear sweep voltammetry (LSV) curves were conducted to evaluate the activity toward HER at a scan rate of 5 mV s^−1^ with *iR* correction. The mass loading of HEA-NPs on GO was 3 wt%. **b** Chronopotentiometry curve for FeCoPdIrPt@GO. **c** The amount of H_2_ during HER.

Our FMBP strategy can facilely produce various HEA-NPs, which have many potential applications. As a proof concept, we applied FeCoPdIrPt@GO HEA-NPs (Supplementary Figs. 29–33) loaded on carbon paper (CP) as the working electrode for hydrogen evolution reaction (HER) in the solution containing KOH (1 M). In order to prove FeCoPdIrPt@GO prepared by FMBP possess high performance toward HER, the single-metallic (e.g., Fe@GO), bi-metallic (e.g., CoPd@GO), tri-metallic (e.g., PtPdIr@GO) samples, even the FeCoPdIrPt@GO prepared by FBP were employed for HER (Supplementary Fig. 32). Through comparison these samples, FeCoPdIrPt@GO (FMBP) exhibited the best performance toward HER. As shown in Fig. 7a, the FeCoPdIrPt@GO exhibited a low overpotential (*ɳ*) of 42 mV at the current density of 10 mA cm^−2^, which was much lower than that of the commercial Pt/C (*ɳ*_10_ = 64 mV). Besides that, at the *ɳ* = 100 mV, the FeCoPdIrPt@GO exhibited with a mass activity of 9.1 mA μg_Pt_, which was 26 times higher than 0.35 mA μg_Pt_ for Pt/C. Moreover, it should be noted that FeCoPdIrPt@GO possessed the superior performance as compared with other electrocatalysts (Supplementary Table 9). The value of *R*_ct_ (charge transfer resistance) for FeCoPdIrPt@GO was much smaller than that of Pt/C (Supplementary Fig. 32), revealing a much more relatively rapid charge transfer between the interface for FeCoPdIrPt@GO and the electrolyte, which boosted the HER activity. In order to confirm the excellent performance of FeCoPdIrPt@GO toward HER, the electrochemical active surface area (ECSA) was also analyzed, calculating from *C*_dl_ (double-layer capacitance), which was ascribed to CV (cyclic voltammetry) curves tested in the non-Faradaic region (Supplementary Fig. 33a). The ECSA for FeCoPdIrPt@GO reached 1462.5 mA cm^−2^ (*C*_dl_ = 58.5 mF cm^−2^), demonstrating that the larger ECSA also contributed to the high HER performance of FeCoPdIrPt@GO. The Tafel slopes of different samples composed of various metals were calculated to investigate the mechanism of HER (Supplementary Fig. 32e). It was seen that the Tafel slopes of Ir and CoPd were 117 and 204 mV dec^−1^, respectively, indicating the limiting step for these samples was the Volmer step (water dissociation)^45^. In contrast, the Tafel slopes of Pt, PtPdIr, and FeCoPtPdIr were decreased to 98, 97, and 82 mV dec^−1^, respectively, indicating the limiting steps of HER on Pt-based alloys were Heyrovsky-Tafel steps (adsorption/desorption of hydrogen species)^45^. FeCoPtPdIr possessed the smallest Tafel slope, indicating the fastest kinetic toward HER. Furthermore, the Faraday efficiency for FeCoPdIrPt@GO is 99.4%, indicating the current mainly came from HER instead of side reactions (Fig. 7c). The excellent performance of FeCoPdIrPt@GO toward HER could be ascribed to the electronic effects of homogenous alloys, due to the synergic effect of HEAs so called “cocktail effect”^19,46^. In addition, multi-element interactions can lead to a huge divergence of the properties and states of the center atoms as compared to atoms in the single-element material^26,46^. According to the theory of electrochemical HER, the free energy for hydrogen species (Δ*G*_H*_) on catalysts close to 0 V would optimize the HER activity due to the lower reaction barrier, which resulted from the balance of adsorption and desorption^47^. Therefore, the compromise between Co (strong adsorption) and Ir (weak adsorption) would moderate Δ*G*_H*_ of FeCoPdIrPt. In addition, although Pd itself has the poor HER activity, it is beneficial to modulate the hydrogen binding energy (HBE) on the Pt surface^47^, improving the HER activity. Moreover, based on the theoretical simulations, the electronic structure (i.e. d-band center) is an important indicator of the activity of electrocatalyst, because the d-orbital electrons determine both bond formation (Δ*G*_H*_) and bond-breaking^48,49^. If the d-band center was too close to or far from the Fermi level, the electrocatalytic activity of catalysts would be reduced. For FeCoPdIrPt, the transition metals (Fe and Co)^50,51^ and Pd^52^ could downshift the antibonding states of Pt, and more electrons would fill the antibonding states, which facilitated the desorption of hydrogen species accelerating hydrogen evolution. Therefore, the tuned d-band center of FeCoPdIrPt contributed to its enhanced HER activity. These results reveal that the FeCoPdIrPt@GO is a highly active and stable catalyst, possessing a promising potential application in practical and commercial conditions. The stability test for FeCoPdIrPt@GO was performed with chronopotentiometry under the same conditions. As shown in Fig. 7b, FeCoPdIrPt@GO exhibited a long stability of 150 h at 10 mA cm^−2^ without obvious change. Simultaneously, FeCoPdIrPt@GO also could run for 150 h at 100 mA cm^−2^ with a decreasing overpotential of 46 mV (Supplementary Fig. 33c). Moreover, the morphology and composition of FeCoPdIrPt on GO maintained well after the HER test (Supplementary Fig. 34). The high stability of the quinary HEA-NPs toward HER was related with entropic stabilization, which leads the thermodynamic stable state, thus, preventing adequate driving forces from degradation of HEA-NPs^53^.

## Discussion

In summary, we develop a facile synthesis strategy, i.e. fast moving bed pyrolysis, for synthesizing the ultrasmall homogeneous HEA-NPs with up to ten elements (MnCoNiCuRhPdSnIrPtAu) highly dispersed on various granular supports. Our strategy can ensure the mixed metal precursors to be simultaneously pyrolyzed at high temperatures, which results in the high supersaturation of monomers and the small size of nuclei, producing the highly dispersed HEA-NPs on supports. The parameters and mechanism of fast moving bed pyrolysis for producing HEA-NPs are carefully investigated. The representative FeCoPdIrPt HEA-NPs exhibit the smaller overpotential and the higher mass activity of HER in electrochemical water splitting as compared with the commercial Pt/C. The enhanced HER performance is attributed to the synergic effect of elements in HEA-NPs. Further, we propose a scalable method for production of supported HEA-NPs (Supplementary Fig. 35). The resultant HEA-NPs holds a promising future for the applications in catalysis, chemical sensing, biology, and energy conversion. Our FMBP strategy opens a venue for synthesizing alloys and nanomaterials with tunable compositions, which have a wide of potential applications.

## Methods

### Materials and reagents

Graphene oxide (GO, thickness, 0.8–1.2 nm) was purchased from Nanjing XFNANO Materials Tech. Co., Ltd and employed as the support. 1,10-Phenanthroline(C_12_H_8_N_2_, 97.0%), Zeolite (Permutit) and γ-Al_2_O_3_ were all purchased from Aladdin. Carbon black VX 72 was bought from Cabot Corporation. Acetone, Cobalt(II) chloride hexahydrate (CoCl_2_·6H_2_O, 99.0%), Copper(II) chloride dihydrate (CuCl_2_·2H_2_O, 99.0%), Manganese(II) chloride tetrahydrate (MnCl_2_·4H_2_O, 99.0%), and Palladium(II) chloride (PdCl_2_, 99.99%) were received from Sinopharm Chemical Reagent Co., Ltd. Rhodium(III) chloride trihydrate (RhCl_3_·3H_2_O, 98%), Chloroauric acid (HAuCl_4_), and Chloroplatinic acid hexahydrate (H_2_PtCl6·6H_2_O, 99.99%) were purchased from Shanghai Macklin Biochemical Co., Ltd. Nickel chloride hexahydrate (NiCl_2_·6H_2_O, 99.999%), Stannic chloride hydrated (SnCl_4_·5H_2_O, 99.995%), and Iridium chloride (IrCl_3_, 99.8%) were received from Aladdin. All the reagents were used without further purification. Ultrapure Milli-Q water (18.2 MΩ cm^−1^) was used to prepare all the aqueous solutions and wash samples. Ethanol was received from Shanghai Lingfeng chemical regent Co., Ltd. Pt/C (20 wt%) was purchased from MACKLIN Biochemical Technology Co., Ltd.

### Supporting metal precursors

Before the preparation of the metal precursors loading on GO (zeolite, γ-Al_2_O_3_, and carbon black), the GO was suspended into ultrapure Milli-Q water and ethyl alcohol by ultrasound for 12 h under the recycled water, maintaining the temperature at 318 K. Then, different metal chlorides were sequentially added to the ultrasonically GO solution containing a certain amount of Phen in order of the metal activity (the order was listed as follows: MnCl_2_·4H_2_O > CoCl_2_·6H_2_O > NiCl_2_·6H_2_O > CuCl_2_·2H_2_O > RhCl_3_·3H_2_O > PdCl_2_ > SnCl_4_·5H_2_O > IrCl_3_ > H_2_PtCl_6_·6H_2_O > HAuCl_4_). Each metal chloride was added into the mixing solution at an interval of 15 min, avoiding the active metal salt being directly reduced. Simultaneously, the aqueous solution was evaporated to dryness at 323 K in the ultrasound system.

### The FMBP strategy for preparation of supported HEA-NPs

We synthesized the HEA-NPs via a fast moving bed pyrolysis (FMBP) strategy. The loading of the HEA-NPs on the supports was 3 wt% for preparation of the ultra-small HEA-NPs. Before calcination, the quartz boat cleaned by ultrasonication in acetone and ethanol for 15 min, respectively. Besides that, the processed quartz boat was dried in vacuum drying oven for 2 h at 355 K. Firstly, the prepared metal precursors loading on GO was evenly placed in a quartz boat, which was placed in a region outside of the furnace about 20 cm.

Afterwards, a vacuum pump was used to extract the gas for 30 min to make the pressure gauge display below 100 Pa. Then, the switch for Ar gas (99.999%) is turned on, keeping flowing Ar (100 sccm) for 30 min to purge the tube. Secondly, the furnace rises to 923 K with a heating rate of 10 K min^−1^. The quartz boat supporting the samples was then pushed into the center of the hot zone with a speed of 20 cm s^−1^ for FMBP (The quartz boat could reach the center of the hot zone within 1 s). After annealing for 120 min at 923 K, the furnace was cooled naturally to the room temperature. Finally, the HEA-NPs can be obtained. Similarly, when Zeolite and γ-Al_2_O_3_ were respectively used as supports, H_2_ (100 sccm) was employed as the reduction gas in the FMBP strategy.

### The FMBP strategy for preparation of the HEA-NPs for EDX

The method for preparation of HEA-NPs for elemental maps was similar to the method for preparation of the ultrasmall HEA-NPs. Except that, the loading of alloy on the supports was 10 wt% for elemental maps.

### The FBP strategy for synthesizing phase-separated NiPdPt

The method for preparation of phase-separated HEA-NPs was different with FMBP and SMBP strategies. The difference among them is in the annealing process. The metal precursors in porcelain boat was heated to 923 K (~31 min) with a rate of 20 K min^−1^ under the flowing of Ar (100 sccm) and maintained 120 min. Then, the furnace was also naturally cool to room temperature. After that, the phase-separated HEA-NPs were obtained.

### The SMBP strategy for preparation of phase-separated NiPdPt

The method for preparation of phase-separated HEA-NPs was similar to that of the FMBP. The difference between two methods is in the annealing process. The metal precursors in quartz boat was pushed into the furnace with a speed of 1 cm s^−1^ (20 s) and heated at 923 K for 2 h under the flowing of Ar (100 sccm). Then, the furnace was also naturally cool to room temperature. After that, the phase-separated HEA-NPs were obtained.

### Simulating temperature variation of supports/precursors

The time for precursors@GO reaching 923 K or 1173 K in the FMBP strategy was simulated with the ANSYS FLUENT software. Details:

Geometry: wall-in (mm) = 350, wall-out (mm) = 150; wall-heat (mm) = 250, Inner diameter of tube (mm) = 23; Density (kg m^−3^): GO = 100, zeolite = 640, Al_2_O_3_ = 460, Quartz = 2650; Mesh number: 1154045.

Specific heat capacity (J kg^−1^ K^−1^): GO = 700, zeolite = 950, Al_2_O_3_ = 840, Quartz = 800.

Coefficient of thermal conductivity (W m^−1^ K^−1^): GO = 5300, zeolite = 0.15, Al_2_O_3_ = 30, Quartz = 2.

Boundary conditions: wall-out = 298 K, wall-in = 298 K, wall-heat = 923 K or 1173 K, velocity magnitude of gas (m s^−1^) = 0.004; Time step size = 0.2 s, number of time steps = 50.

### Physical and chemical characterizations

#### XRD characterization

X-ray powder diffraction (XRD) patterns for HEA-NPs were characterized by a Rigaku D/Max 2400 X-ray diffractometer furnishing with Cu K*α* radiation (*λ* = 1.5406 Å) with a scan rate of 5° min^−1^. All the patterns were recorded in the range of 30–90° (2-Theta).

#### XPS characterization

The patterns of X-ray photoelectron spectroscopy (XPS) for the samples were all performed on a Kratos Axis Ultra DLD using Mg Kα as the excitation source.

#### TEM characterization

The morphology and microstructure of the multimetallic alloy were characterized by an atomic resolution analytical transmission electron microscopy (TEM) (Titan G2 80–200 ChemiSTEM, FEI worked at 200 kV and equipped with 4 probe super EDS) featuring with high-resolution TEM (HRTEM), high-angle annular dark-field scanning transmission electron microscopy (HAADF-STEM), and corresponding energy-dispersive X-ray (EDX) spectrometry. The samples were prepared via dropping the HEA-NPs, which were dispersed in ethanol, onto the carbon-coated molybdenum TEM grids employing capillary at least five times.

#### ICP characterization

The HEA-NPs loading was determined with inductively coupled plasma (ICP) analysis carried out on an Agilent ICP-OES730 instrument. The quinary (CuSnPdPtAu), octonary (CoNiCuPdSnIrPtAu), and denary (MnCoNiCuRhPdSnIrPtAu) alloy were characterized. Firstly, a certain amount of the samples were weighed, then, these samples were added to a mixed solution containing 6 mL of aqua regia and 1 mL of hydrofluoric acid. Then oven was heated to 458 K and keeping for 8 h. After cooling to room temperature, transferring, and testing in a constant volume instrument.

#### The electrochemical test for HER

The HER performance of the prepared electrode was performed on a Bio-Logic VSP potentiostat with a standard three-electrode system. The prepared quinary (FeCoPdPtIr) HEA-NPs supported on GO (3 wt%) were loaded on carbon paper (CP, 1 × 1 cm^2^), which was employed as the working electrode. In order to prove the quinary (FeCoPdPtIr) alloy exhibited an excellent activity toward HER, the pure CP was also applied as the working electrode. The mass of the quinary (FeCoPdPtIr@GO) HEA-NPs on CP was 1 mg cm^−2^. Similarly, the weight of the commercial Pt/C loaded on CP was 1.2 mg cm^−2^. Besides that, the graphite rod and Hg/HgO were used as the counter and reference electrodes, respectively. The LSV curves of the prepared samples were conducted in a 1-M KOH solution (pH = 13.8). Simultaneously, the obtained potentials were all calculated with *iR* correction and transformed into reversible hydrogen electrode (RHE). All the electrochemical impedance spectroscopy (EIS) tests for these samples were conducted at the onset potential from 0.01 Hz to 100 kHz. The stability for the prepared quinary (FeCoPdPtIr) alloy was carried out 1 M KOH solution at a constant current density of 10 mA cm^−2^ toward HER. Moreover, the commercial Pt/C (20%, 1 mg cm^−2^) were used toward HER as the compared test. The double layered capacitances (*C*_dl_) of the working electrodes were calculated by CV curves from the potentials (0.11–0.21 V vs RHE) with different scan rates (10–30 mV s^−1^). The current densities were linear with the scan rates, and the slope was *C*_dl_. In order to calculate ECSA, the applied the specific capacitance (20–60 μF cm^−2^) was set as 40 μF cm^−2^.

6 $${\mathrm{ECSA}} = C_{{\mathrm{dl}}}/C_{\mathrm{s}} \times {\mathrm{ASA}}$$

Where *C*_s_ is the specific capacitance and ASA is the actual surface area of substrates.

#### Calculation of Faradaic efficiency

The Faradaic efficiency (FE) for quinary (FeCoPdPtIr) alloy in 1 M KOH solution during HER was conducted in a three-electrode configuration. The amount of H_2_ during reaction was detected by a gas chromatography (GC, 9790II, Hangzhou Gatai Scientific Instruments), using the thermal conductivity detector. Before test, the electrolyte (under stirring) and cell were first degassed to remove air with Argon for 30 min at least. A constant current of 10 mA was used for producing hydrogen. The gaseous samples were drawn with a gas tight syringe, and the amount of hydrogen was obtained the GC instrument. The gases were drawn and analyzed three times and the average value of them was used.

The Faradaic efficiency (FE) of HER on electrodes is calculated with the equation:

7 $${\mathrm{FE}}\left( {{\mathrm{H}}_2,{\mathrm{\% }}} \right) = \frac{{V_{{\mathrm{H}}_2} \times 2 \times F \times i \times t}}{{V_m}} \times 100{\mathrm{\% }}$$

*V*_H2_ is the measured volume of H_2_, *F* is the Faraday constant (96,485 C mol^−1^), *i* is the current, *t* is the time for electrolysis for producing hydrogen, and *V*_*m*_ is the molar volume of the gas.

## Supplementary information

Supplementary Information

Description of Additional Supplementary Files

Supplementary Movie 1

Supplementary Movie 2

## Data Availability

All source data supporting the findings of this study are available from the corresponding authors upon reasonable request.
