# Supplementary material for: Synthesis of high-entropy alloy nanoparticles on supports by the fast moving bed pyrolysis
Source: Nat Commun. 2020 Apr 24;11:2016. doi: 10.1038/s41467-020-15934-1 (PMC7181682; doi:10.1038/s41467-020-15934-1)
Supplement: Supplementary file 1 — Supplementary Information [file 41467_2020_15934_MOESM1_ESM.pdf]

Supplementary Information

Synthesis of high-entropy alloy nanoparticles on supports by the fast moving bed pyrolysis

Gao *et al*.

Contents

Supplementary Figure 1 Elemental maps for denary precursors on GO.. 4
Supplementary Figure 2 Elemental maps for octonary precursors on GO...................................... 5
Supplementary Figure 3 HAADF-STEM images for quinary (CuPdSnPtAu) alloy.................................... 6
Supplementary Figure 4 HAADF-STEM images for octonary (CoNiCuPdSnIrPtAu) alloy........................ 7
Supplementary Figure 5 XRD patterns NiPtPd samples.. 8
Supplementary Figure 6 XRD patterns of alloys containing 5-10 metals by FMBP................................ 9
Supplementary Figure 7 HRTEM images and EDX spectra for the NiPdPt alloy.................................... 10
Supplementary Figure 8 HRTEM images and EDX spectra for (CuPdSnPtAu) alloy.............................. 11
Supplementary Figure 9 HRTEM images and EDX spectra for (NiCuSnPdPtAu) alloy.......................... 12
Supplementary Figure 10 HRTEM images and EDX spectra for (CoNiCuPdSnPtAu) alloy.................. 13
Supplementary Figure 11 HRTEM images and EDX spectra for (NiCuPdSnPtAuIr) alloy...................... 14
Supplementary Figure 12 HRTEM images and EDX spectra for (CoNiCuPdSnIrPtAu) alloy.................. 15
Supplementary Figure 13 HRTEM images and EDX spectra of (MnCoNiCuRhPdSnIrPtAu) alloy.......... 16
Supplementary Figure 14 The atom ratios of metals in 5 (FeCoPdPtIr) nanoparticles............................... 17
Supplementary Table 1-3 ICP analysis of HEA-NPs synthesized by FMBP.. 18
Supplementary Figure 15 XPS spectra for the ternary (NiPtPd) alloy... 19
Supplementary Figure 16 XPS spectra for octonary (CoNiCuSnPdIrPtAu) alloy...................................... 20
Supplementary Figure 17-19 XPS spectra for denary (MnCoNiCuRhPdSnIrPtAu) alloy......................... 21
Supplementary Figure 20 STEM images of alloys containing 5-10 metals by FMBP............................... 24
Supplementary Figure 21 Size distribution of HEA-NPs on GO by FMBP at 923K................................ 25
Supplementary Figure 22 The ternary (NiPdPt) alloy synthesized by FBP at 923 K................................ 26
Supplementary Figure 23 Diagram of free energy change versus the nucleus size (r)............................... 27
Supplementary Figure 24 Time for various supports reached different temperature................................. 27
Supplementary Figure 25 Nucleation rate versus temperature.. 28
Supplementary Note 1 The mechanism of nucleation.. 29
Supplementary Table 4. The surface free energy for GO, γ-Al2O3, and Zeolite... 30
Supplementary Table 5. The molar volume for these employed metal precursors..................................... 30
Supplementary Table 6 Critical radius (r^*) and critical excess free energy (ΔG_{r^*}) at 673 K and 923 K...... 30
Supplementary Figure 26 Elemental maps of NiPdPt by FMBP at different temperature......................... 31
Supplementary Figure 27 STEM images for NiPdPt by FMBP at different temperature............................ 32
Supplementary Figure 28 STEM images for CuSnPdPtAu by FMBP for different time............................ 32
Supplementary Figure 29 Elemental maps for FeCoPdIrPt supported on GO.. 33
Supplementary Figure 30 STEM image for FeCoPdIrPt supported on GO.. 33
Supplementary Figure 31 HRTEM images and EDX spectra for FeCoPdIrPt on GO................................34
Supplementary Table 7 Chemical reduction potentials of the used metal ions... 35
Supplementary Table 8 Physical properties of metals used in this study.. 35

Supplementary Figure 32 Electrochemical performance comparison of samples..36

Supplementary Table 9 Comparison of representative electrocatalysts by different methods for HER performance in alkaline solution.. 37

Supplementary Figure 33 CV curves for the quinary (FeCoPdIrPt) HEA-NPs..38

Supplementary Figure 34 HRTEM images and EDX spectra for FeCoPdIrPt after HER test......................38

Supplementary Figure 35 Scalable production of supported HEA-NPs by FMBP.....................................39

References...40

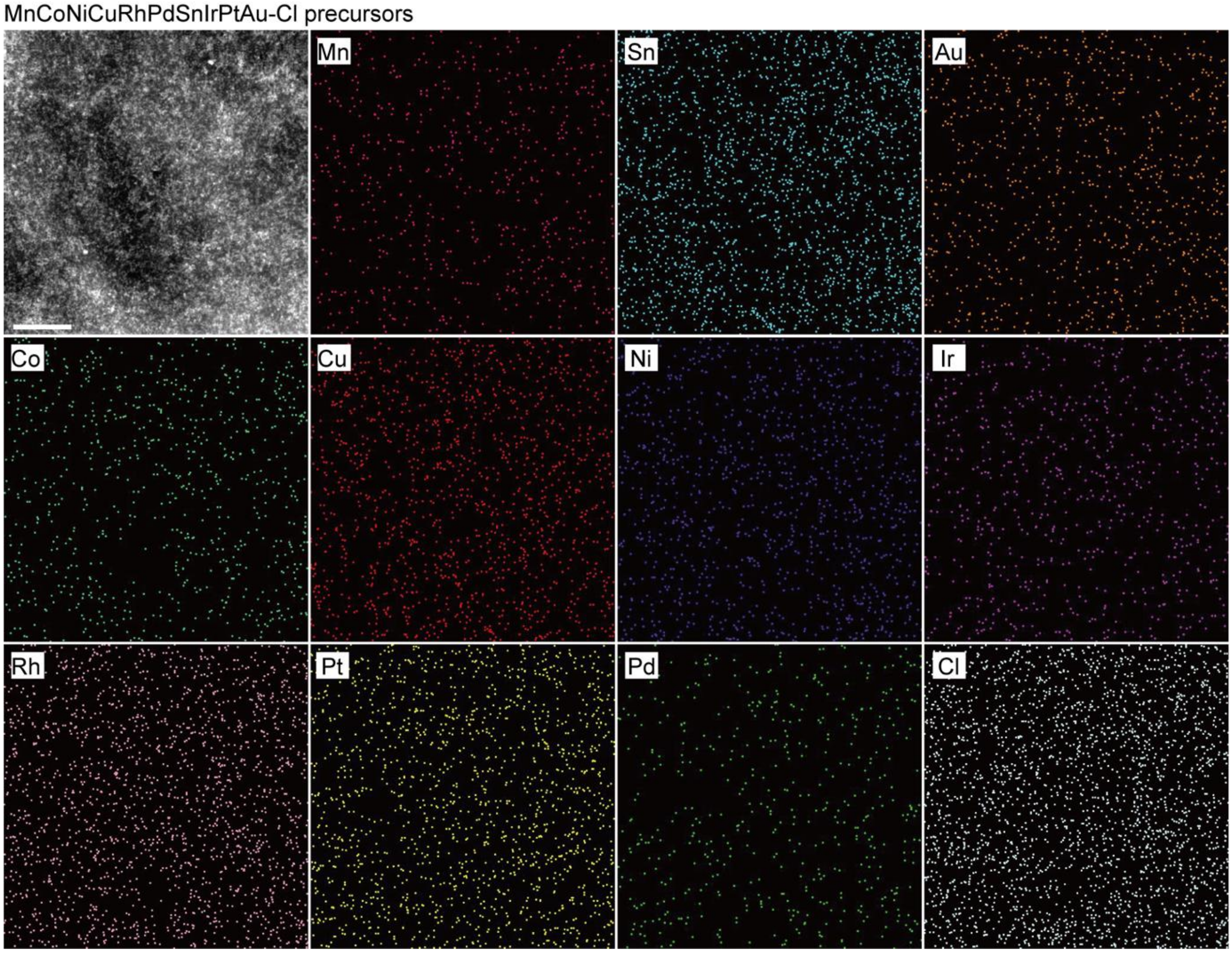

Supplementary Figure 1. Elemental maps for denary precursors on GO. The elemental maps for denary (MnCoNiCuRhPdSnIrPtAu) metal chloride precursors on GO. Scale bar: 90 nm.

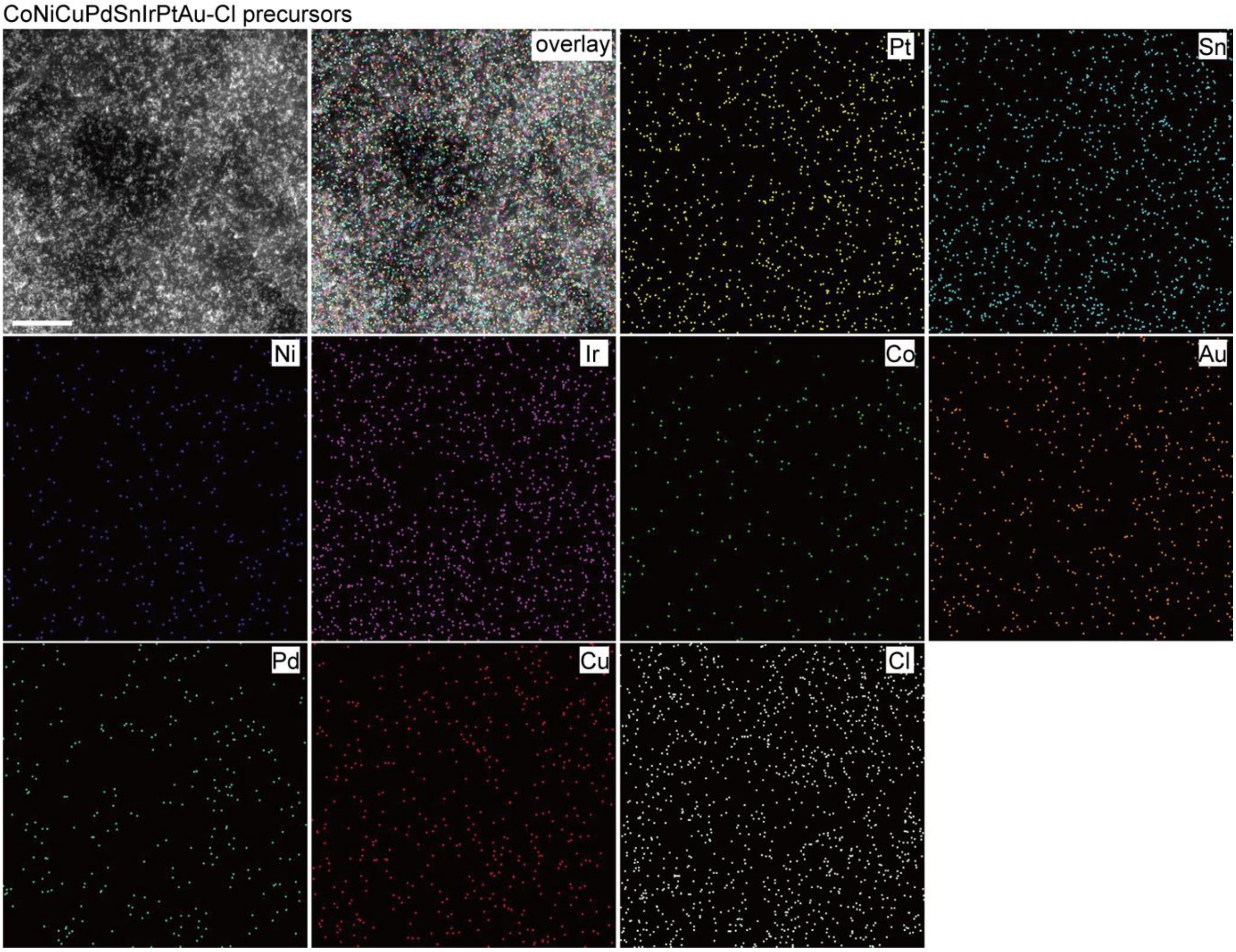

Supplementary Figure 2. Elemental maps for octonary precursors on GO. The elemental maps for octonary (CuCoNiPdSnIrPtAu) metal chlorides precursors on GO. Scale bar: 90 nm.

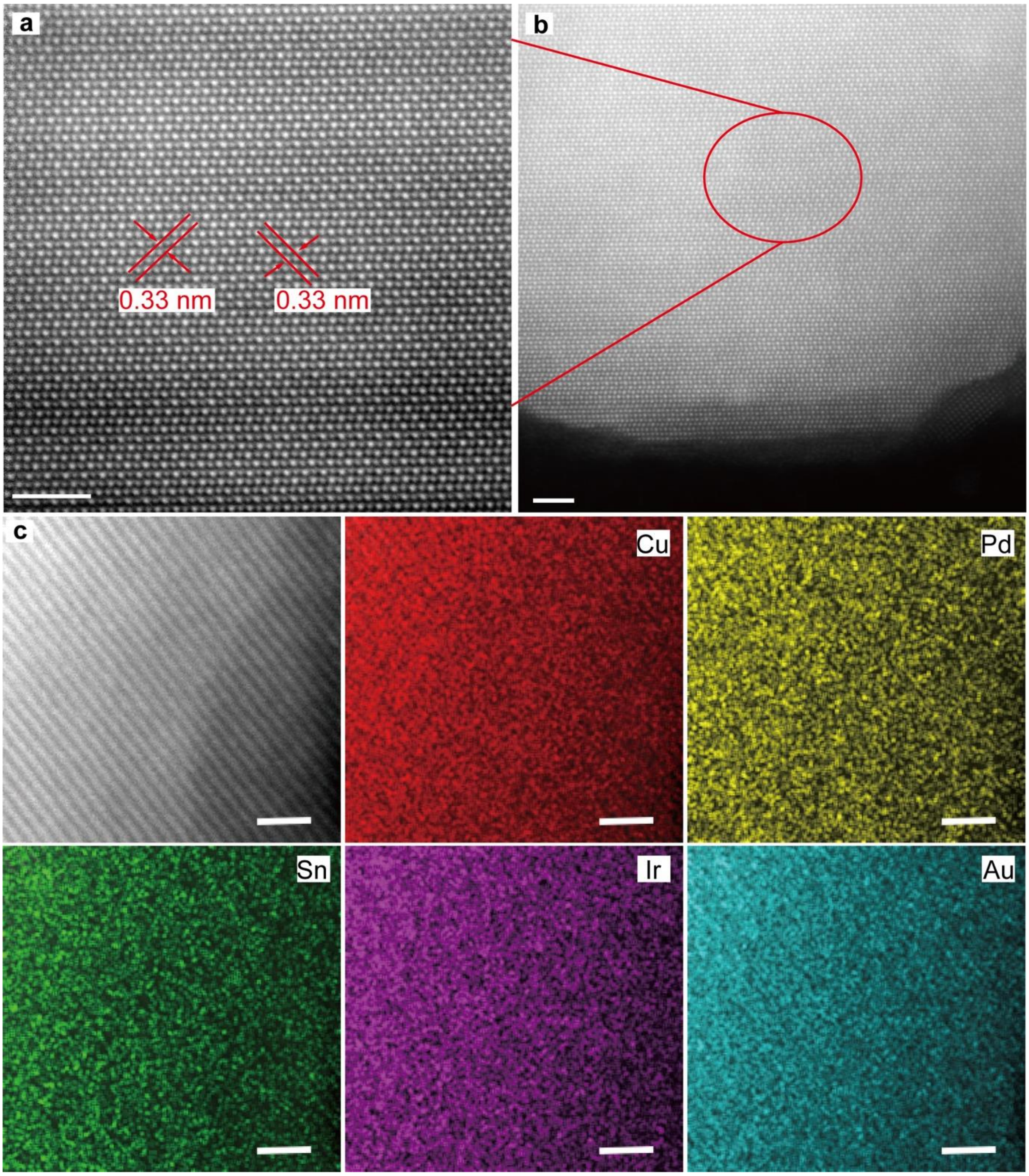

Supplementary Figure 3. HAADF-STEM images for quinary (CuPdSnPtAu) alloy. (**a, b**) HAADF-STEM images for quinary (CuSnPdPtAu) alloy supported on GO at 923 K. a is the partial magnification area of the red circle in b. (**c**) The atomic-scale HAADF-STEM image for CuSnPdAuPt alloy and corresponding elemental maps. The loading of HEA-NPs on GO was 10 wt%. Scale bar (a-b): 2nm, c: 1nm.

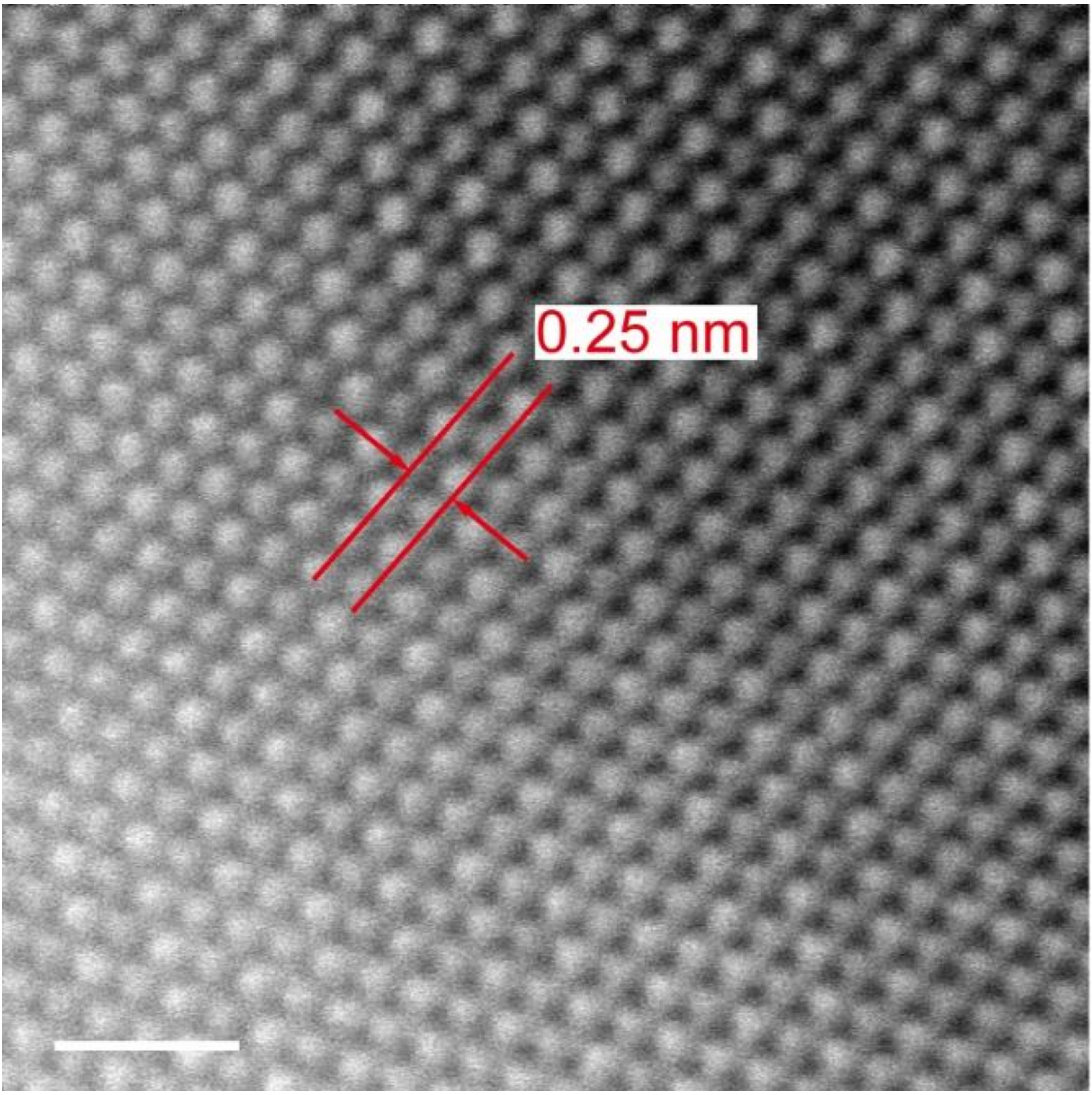

Supplementary Figure 4. HAADF-STEM images for octonary (CoNiCuPdSnIrPtAu) alloy. The octonary (CoNiCuSnPtPdAuIr) HEA-NPs supported on GO at 923 K. The loading of HEA-NPs on GO was 10 wt%. Scale bar: 1 nm.

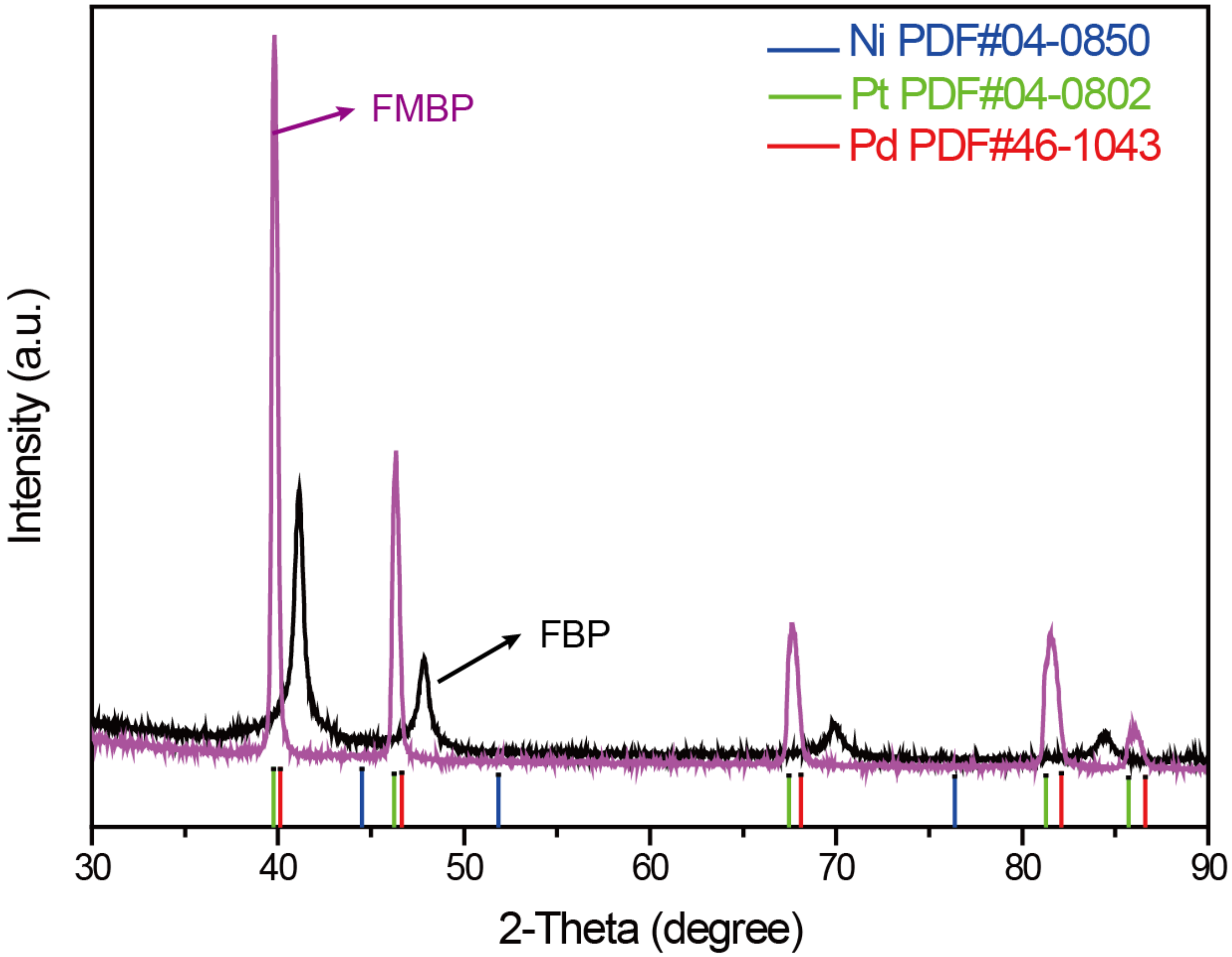

Supplementary Figure 5. XRD patterns NiPtPd samples. The temperature for NiPdPt supported on GO by the FMBP and FBP method was 923 K. The loading of HEA-NPs on GO was 10 wt%.

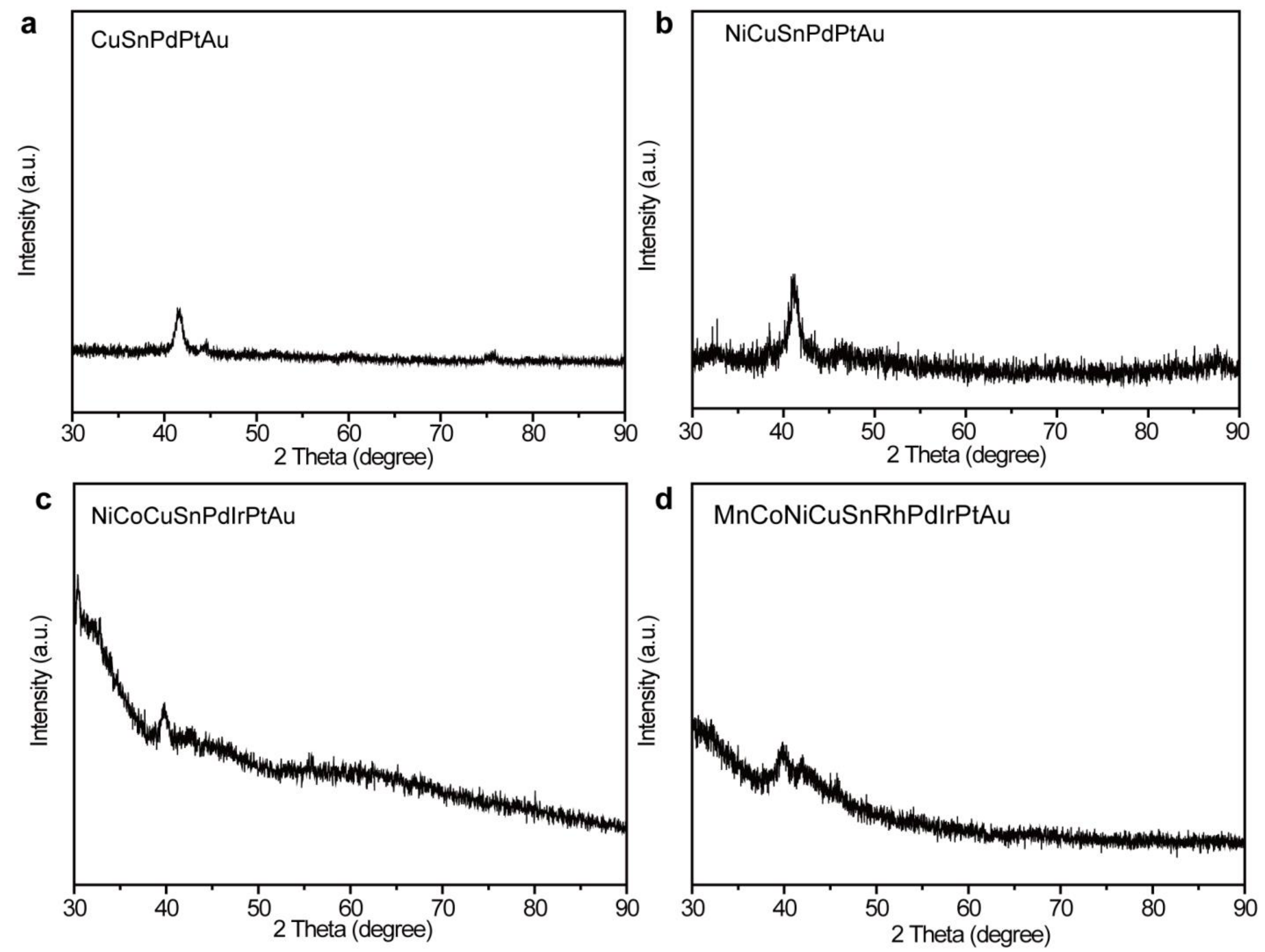

Supplementary Figure 6. XRD patterns of alloys containing 5-10 metals by FMBP. quinary (CuSnPdPtAu) HEA-NPs (**a**), senary (NiCuSnPdPtAu) HEA-NPs (**b**), octonary (NiCoCuSnIrPdPtAu) HEA-NPs (**c**), and denary (MnCoCuIrNiSnRhPdPtAu) HEA-NPs (**d**) by FMBP at 923 K. The loading of HEA-NPs on GO was 10 wt%.

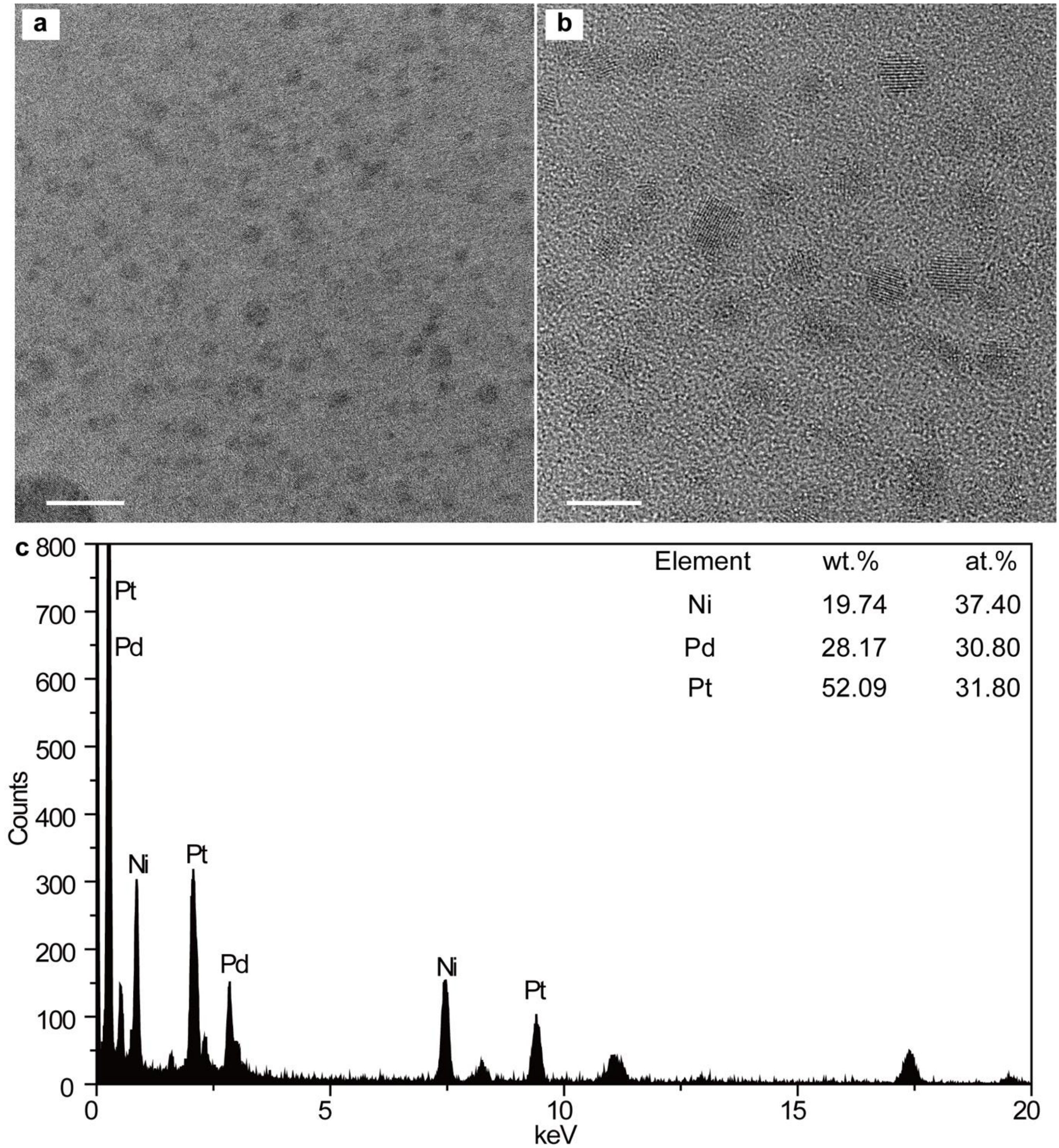

Supplementary Figure 7. HRTEM images and EDX spectra for the NiPdPt alloy. HRTEM images (**a**, **b**) and EDX spectrum (**c**) for the ternary (NiPdPt) alloy supported on GO by the FMBP strategy at 923 K. The loading of HEA-NPs on GO was 3 wt%. Scale bar a: 10 nm, b: 5 nm.

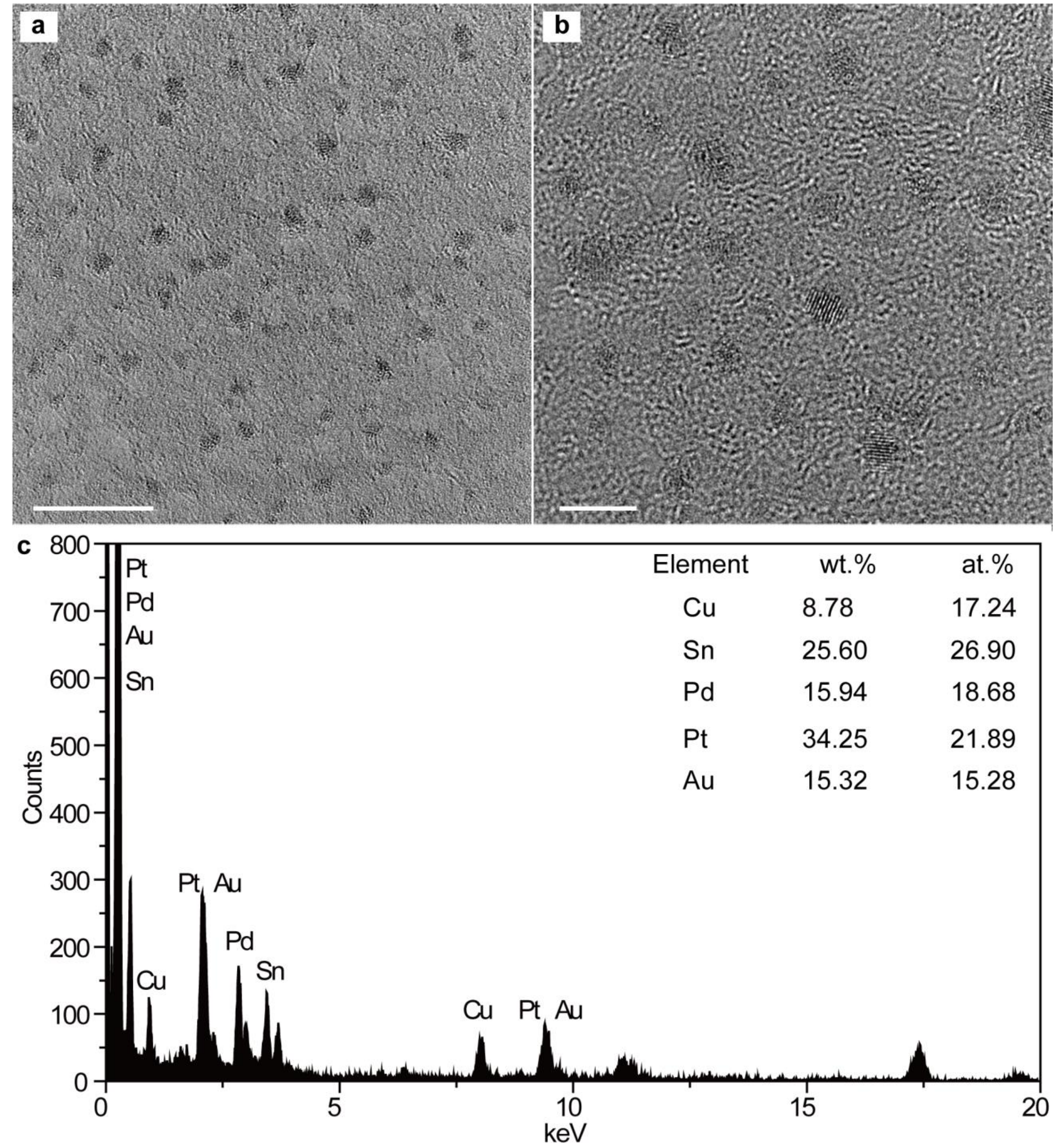

Supplementary Figure 8. HRTEM images and EDX spectra for (CuPdSnPtAu) alloy. HRTEM images (**a, b**) and EDX spectra (**c**) for the quinary (CuPdSnPtAu) HEA-NPs. The loading of HEA-NPs on GO was 3 wt%. Scale bar a: 10 nm, b: 5 nm.

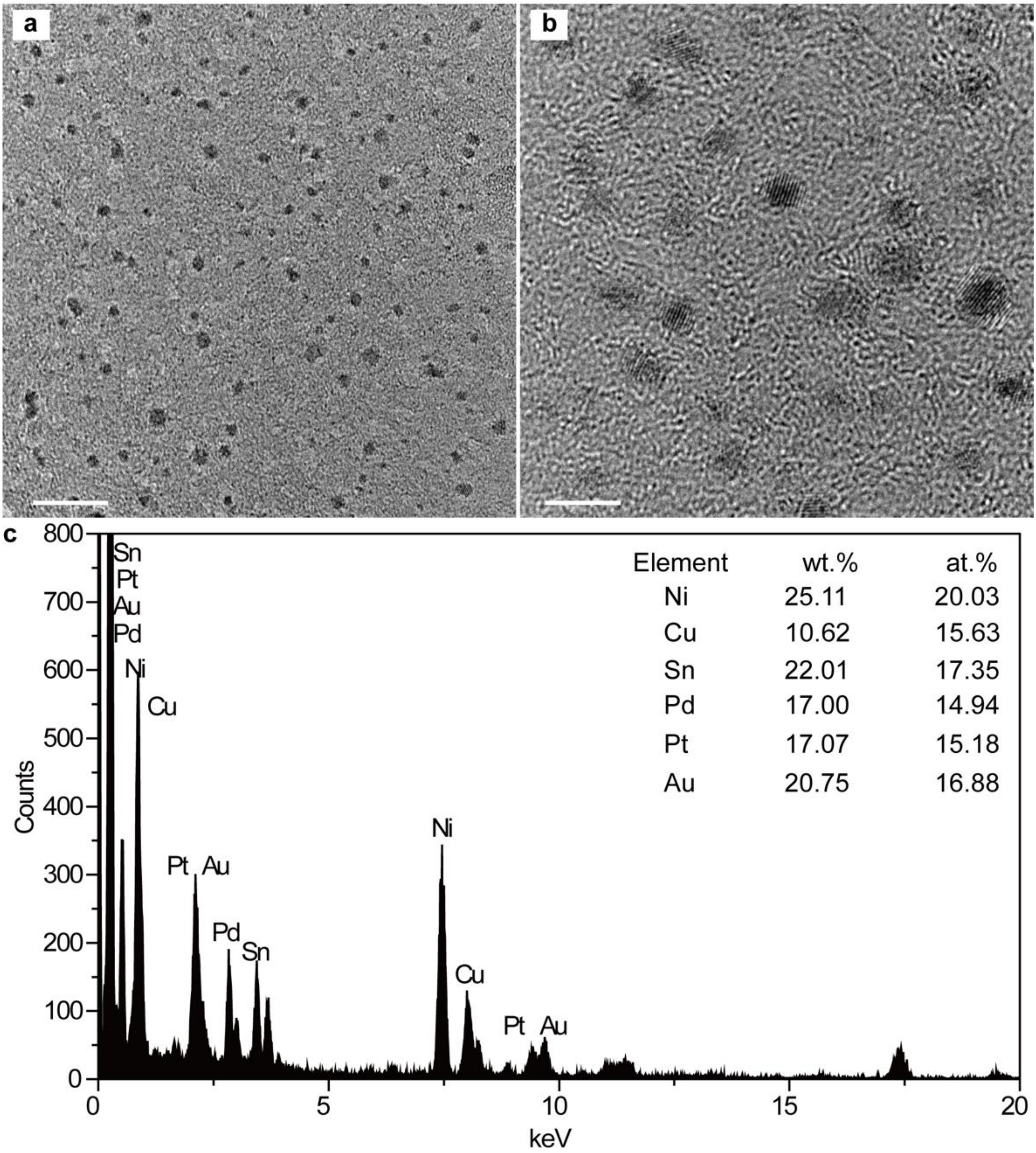

Supplementary Figure 9. HRTEM images and EDX spectra (NiCuSnPdPtAu) alloy. HRTEM images (**a**, **b**) and EDX spectrum (**c**) for the senary (NiCuSnPdPtAu) HEA-NPs supported on GO at 923 K. The loading of HEA-NPs on GO was 3 wt%. Scale bar a: 10 nm, b: 5 nm.

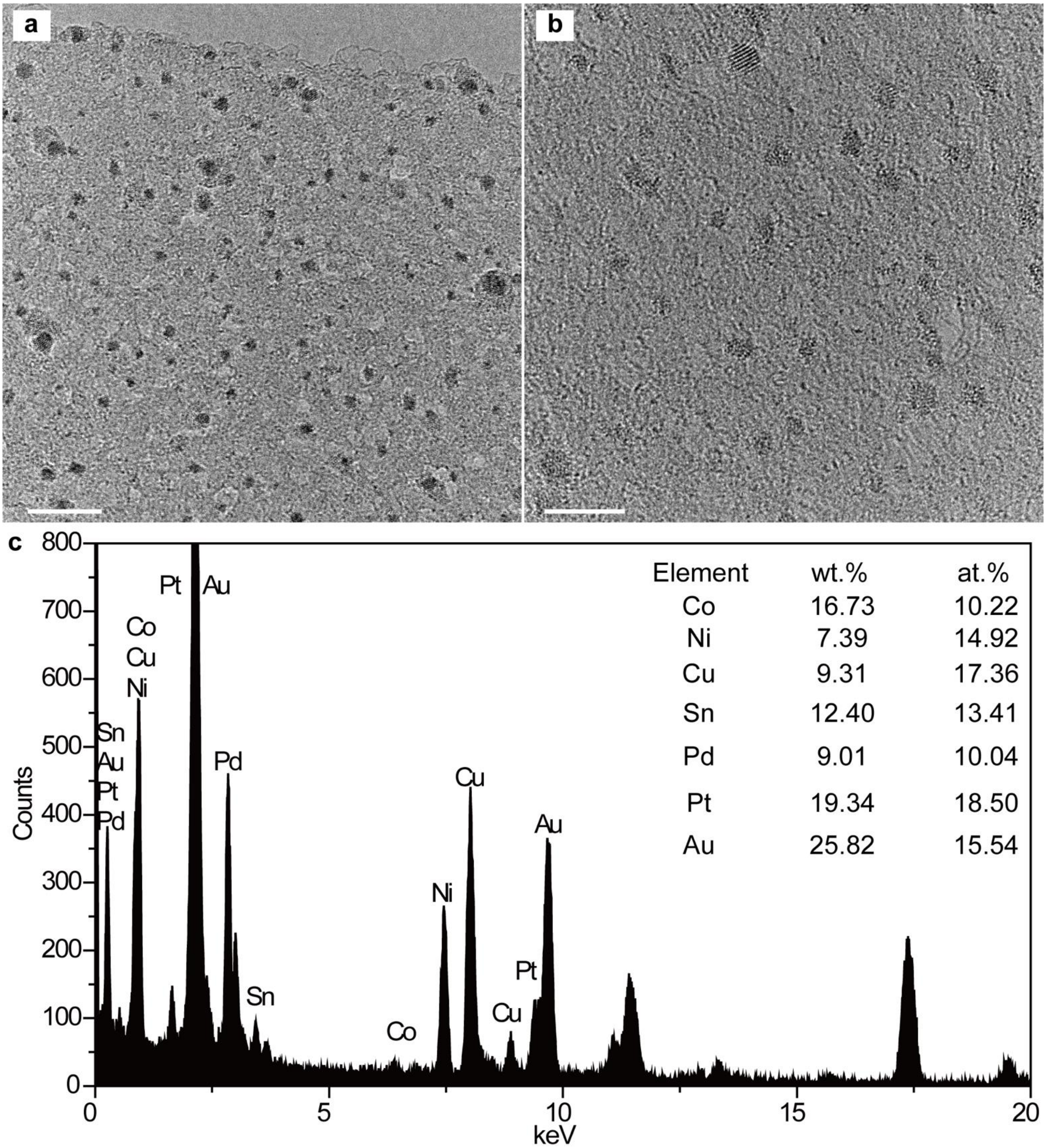

Supplementary Figure 10. HRTEM images and EDX spectra for (CoNiCuPdSnPtAu) alloy. HRTEM images (**a**, **b**) and EDX spectrum (**c**) for the septenary (CoNiCuSnPdPtAu) HEA-NPs supported on GO at 923 K. The loading of HEA-NPs on GO was 3 wt%. Scale bar a: 10 nm, b: 5 nm.

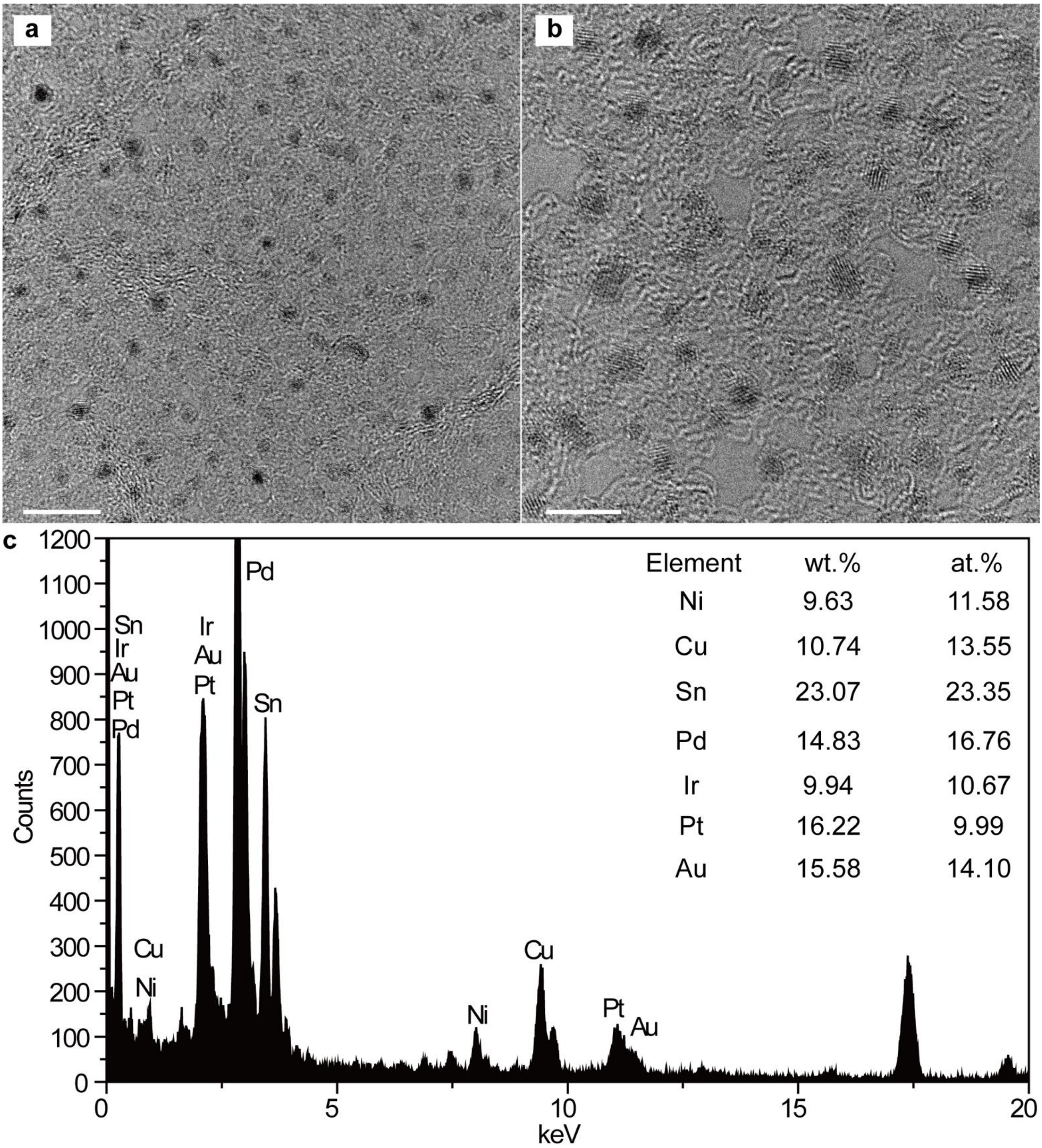

Supplementary Figure 11. HRTEM images and EDX spectra for (NiCuPdSnPtAuIr) alloy. HRTEM images (**a**, **b**) and EDX spectrum (**c**) for the septenary (NiCuSnPdIrPtAu) HEA-NPs supported on GO. The loading of HEA-NPs on GO was 3 wt%. Scale bar a: 5 nm, b: 5 nm.

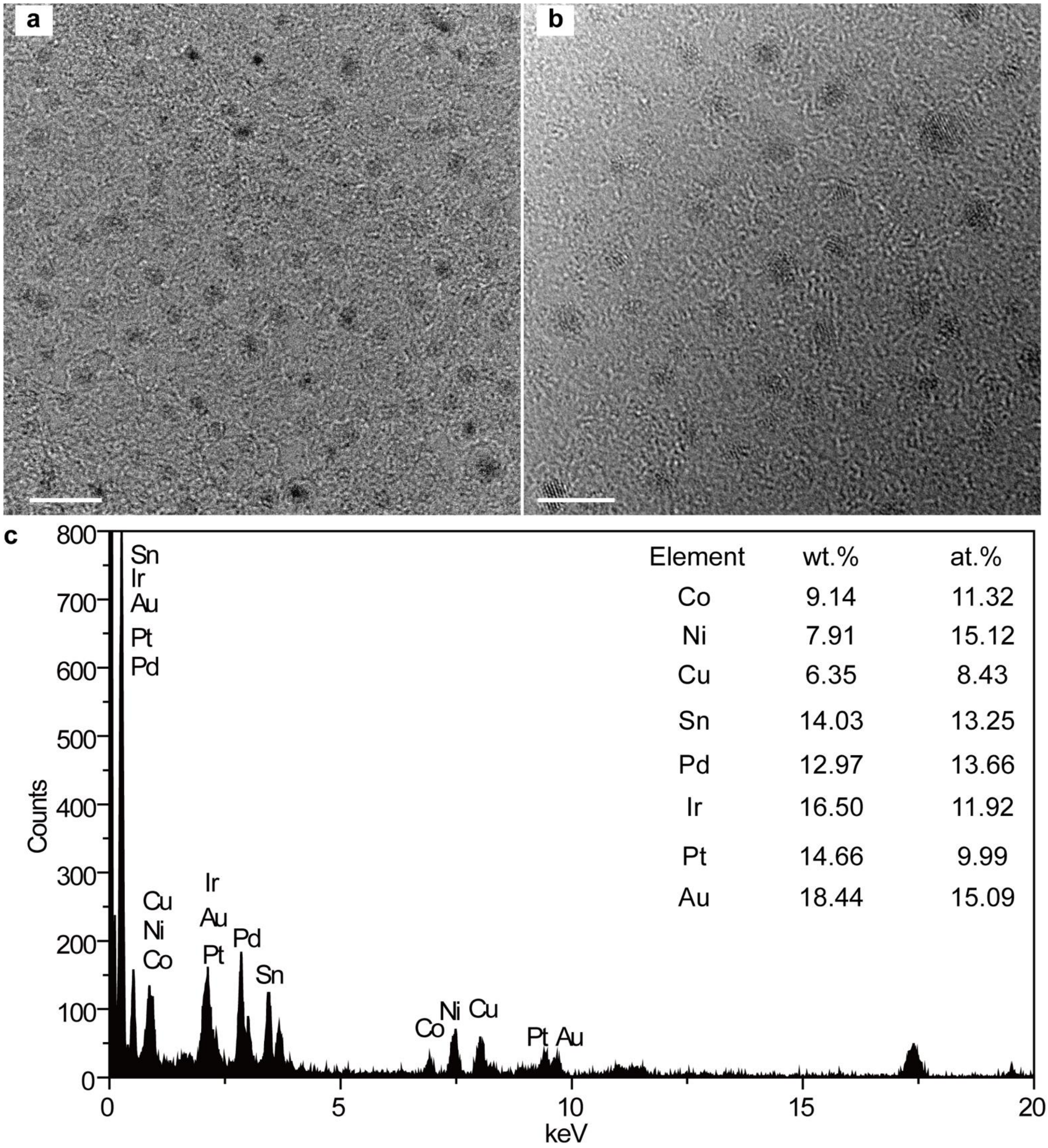

Supplementary Figure 12. HRTEM images and EDX spectra for (CoNiCuPdSnIrPtAu) alloy. HRTEM images (**a**, **b**) and EDX spectrum (**c**) for the octonary (CoNiCuSnPtPdAuIr) HEA-NPs supported on GO at 923 K. The loading of HEA-NPs on GO was 3 wt%. Scale bar a: 10 nm, b: 5 nm.

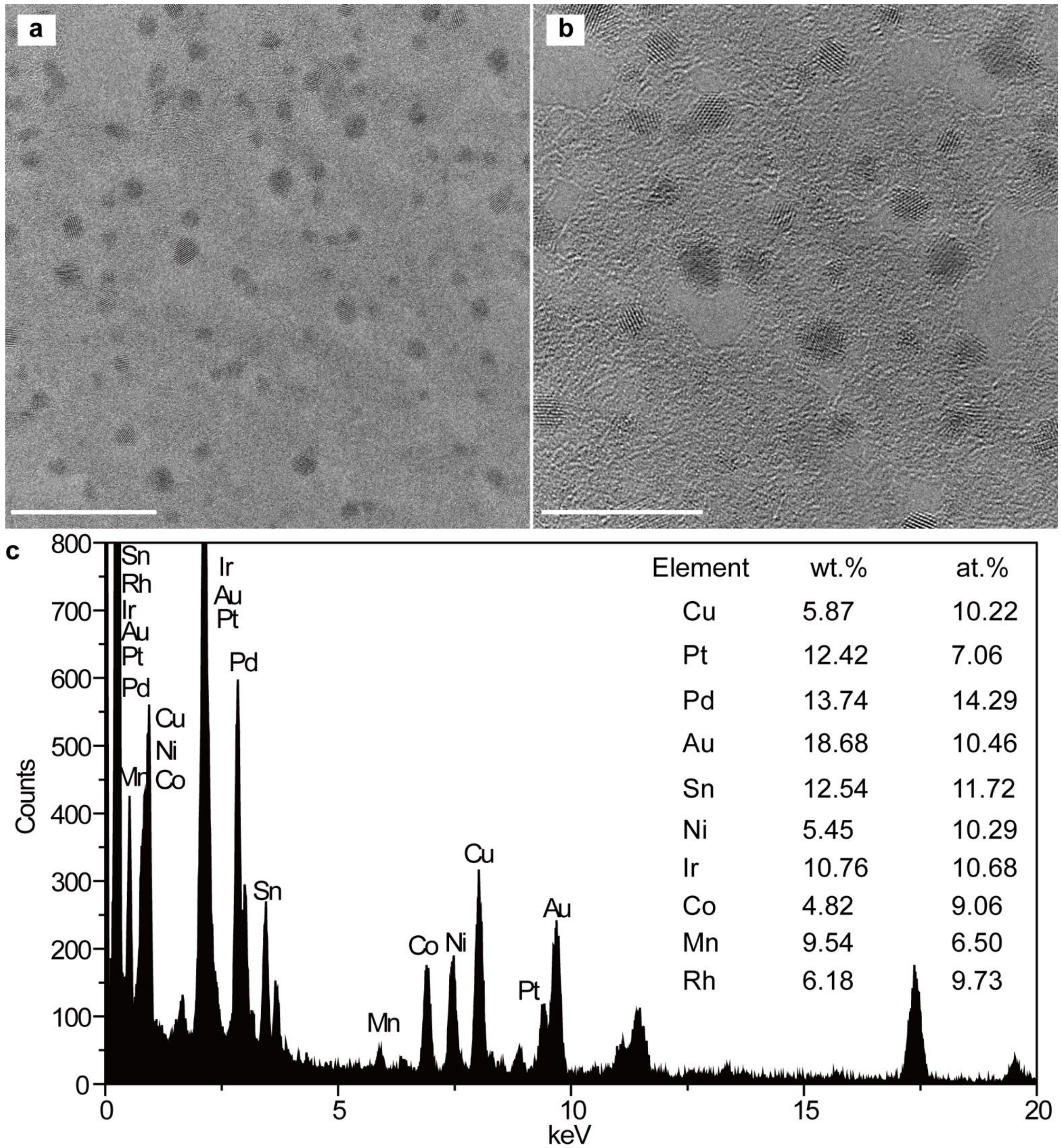

Supplementary Figure 13. HRTEM images and EDX spectra of (MnCoNiCuRhPdSnIrPtAu) alloy. HRTEM images (**a**, **b**) and EDX spectra (**c**) for the denary (MnCoNiCuSnRhPdIrPtAu) HEA-NPs supported on GO at 923 K. The loading of HEA-NPs on GO was 3 wt%. Scale bar a: 20 nm, b: 10 nm.

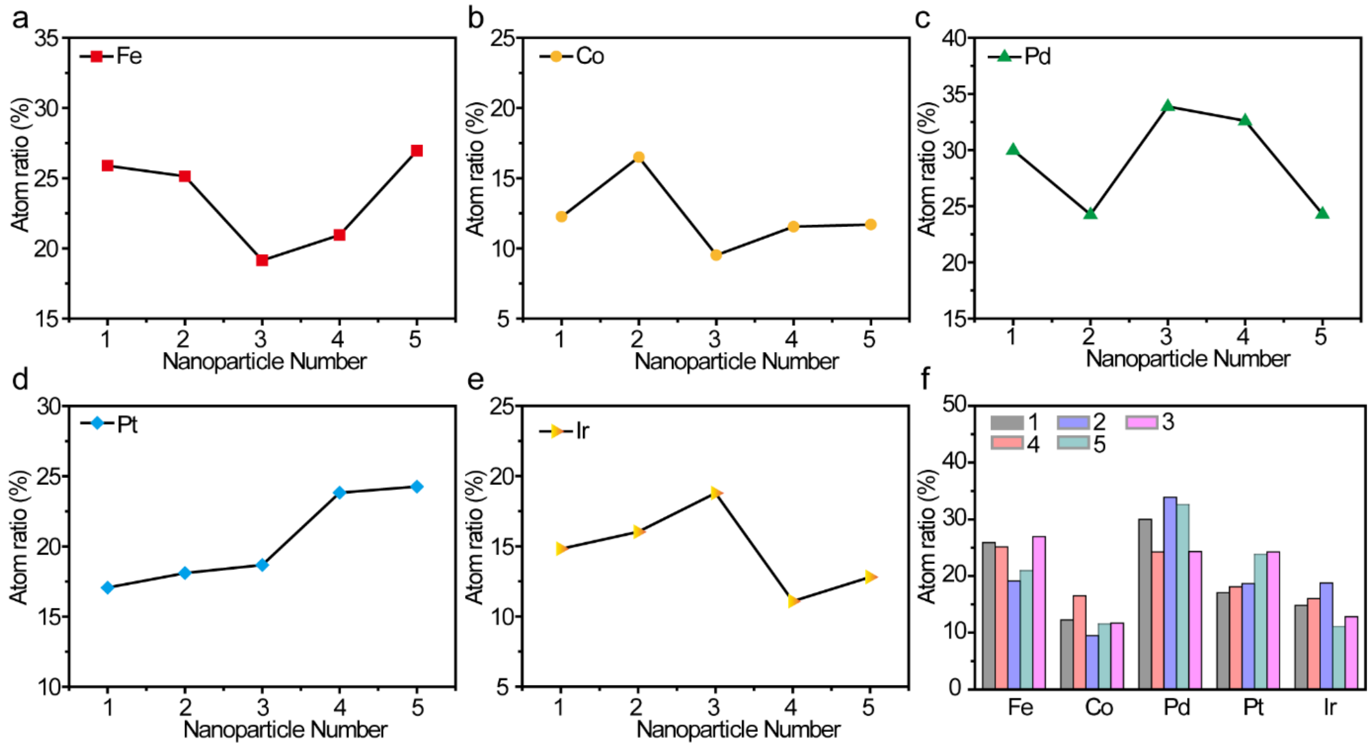

Supplementary Figure 14. The atom ratios of metals in 5 (FeCoPdPtIr) nanoparticles. The atom ratios of Fe (**a**), Co (**b**), Pd (**c**), Pt (**d**), Ir (**e**) in 5 individual nanoparticles of quinary (FeCoPdPtIr) HEA-NPs. (**f**) the column diagram of atom ratios for Fe, Co, Pd, Pt, Ir in 5 individual nanoparticles of quinary (FeCoPdPtIr) HEA-NPs.

ICP analysis of HEA-NPs synthesized by FMBP

Supplementary Table 1. The concentration of each element in the denary (MnCoNiCuSnIrPdPtAuRh) HEA-NPs.

element	Conversion content	at.%
Au	42235.27	1.18
Co	12617.49	1.18
Cu	12968	1.12
Ir	40078.30	1.14
Mn	10280.31	1.03
Ni	10646	1
Pd	23649.94	1.22
Pt	39733.08	1.12
Rh	22940.67	1.22
Sn	24587.83	1.14

Supplementary Table 2. The concentration of each element in the octonary (CoNiCuSnIrPdPtAu) HEA-NPs.

element	Conversion content	at.%
Au	41868.73	1.10
Co	11438.89	1.01
Cu	14239	1.16
Ir	36810.90	1
Ni	11819.8	1.05
Pd	24713.94	1.21
Pt	43904.14	1.17
Sn	26961.83	1.18

Supplementary Table 3. The concentration of each element in the quinary (CuSnPdPtAu) HEA-NPs.

element	Conversion content	at.%
Au	39110.73	1
Cu	13095.1	1.03
Pd	21521.94	1.01
Pt	38831.54	1.00
Sn	24706.53	1.04

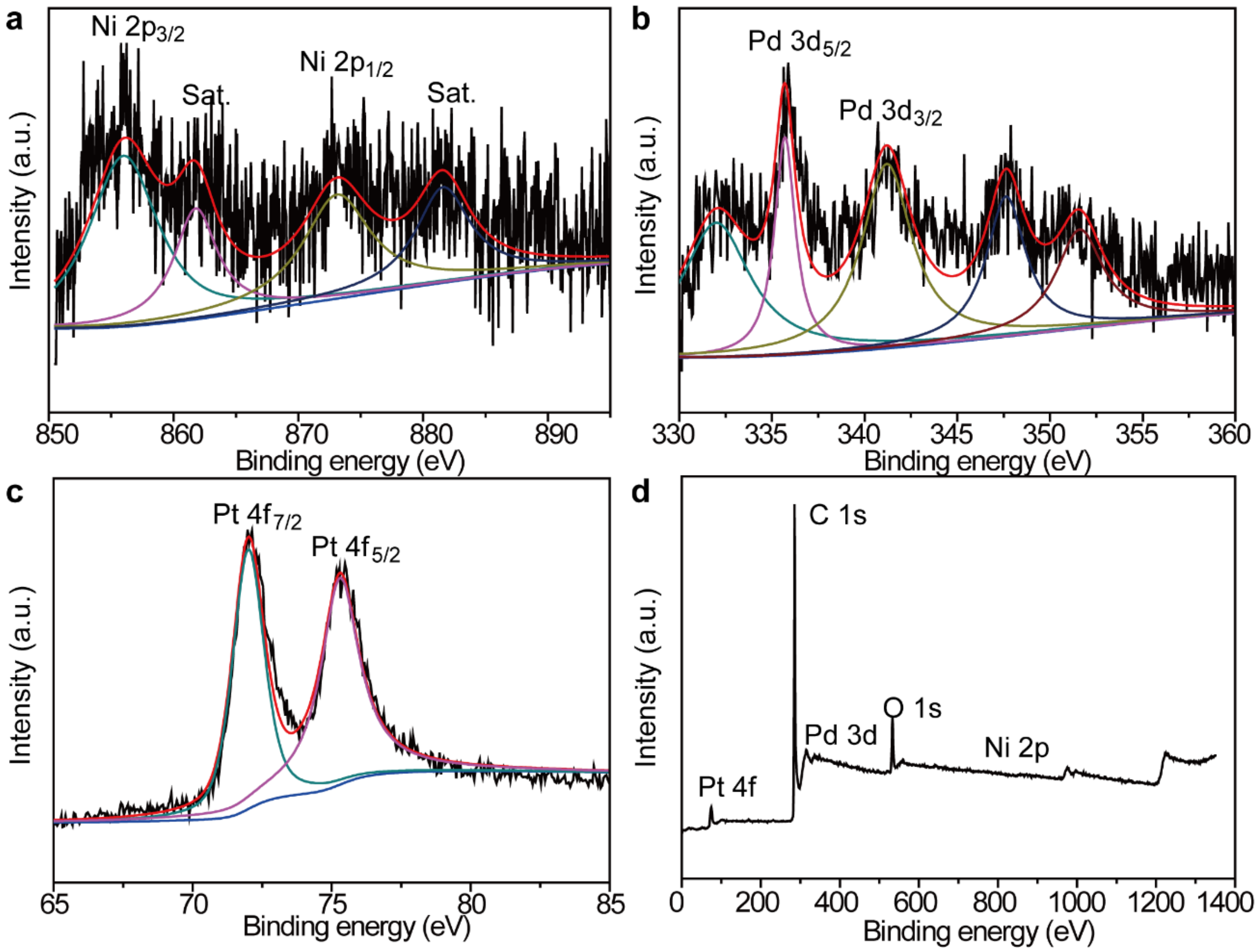

Supplementary Figure 15. XPS spectra for the ternary (NiPtPd) alloy. (**a-c**) The XPS spectra of Ni, Pd, and Pt for the ternary (NiPtPd) alloy, respectively. The loading of HEA-NPs on GO was 3 wt%. Ni (Ni $2p_{3/2}$ - 855.8 eV; Ni $2p_{1/2}$ - 872.6 eV). Pd (Pd $3d_{5/2}$ - 336 eV; Pd $3d_{3/2}$ - 341.3 eV)[1]. Pt (Pt $4f_{7/2}$ - 72.1 eV; Pt $4f_{5/2}$ - 75.3 eV)[2]. The phenomenon of the metallic oxidized state should be ascribed to the metal on the surface of the alloy oxidized by air.

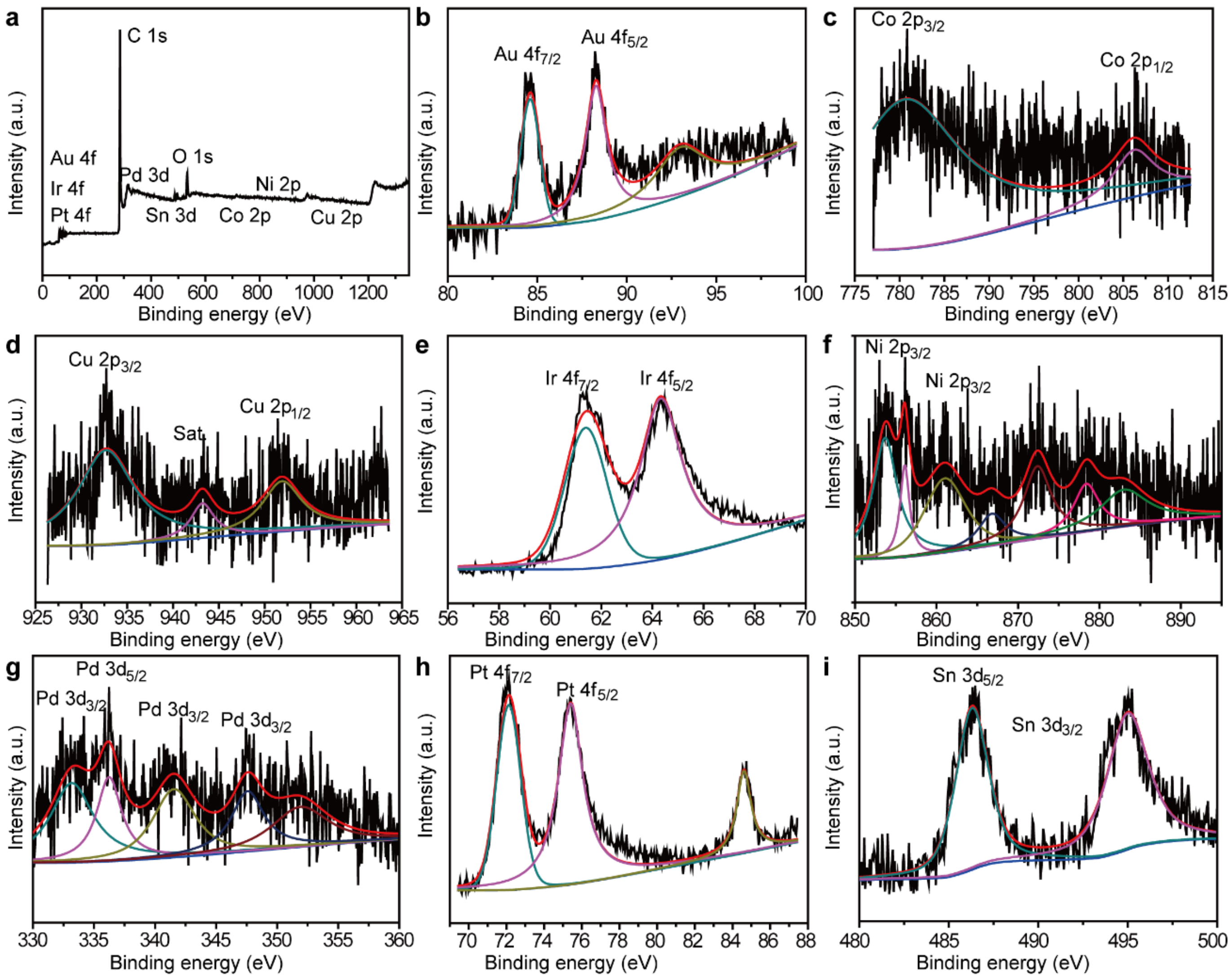

Supplementary Figure 16. XPS spectra for octonary (CoNiCuSnPdIrPtAu) alloy. (**a**) The XPS spectrum for octonary (AuCoCuIrNiPdPtSn) HEA-NPs at 923 K. **(b-i**) The XPS spectra of Au, Co, Cu, Ir, Ni, Pd, Pt, and Sn for the octonary (AuCoCuIrNiPdPtSn) HEA-NPs. The loading of HEA-NPs on GO was 3 wt%. Au (Au $4f_{7/2}$ - 84.5 eV; Au $4f_{5/2}$ - 88.3 eV)[3]. Co (Co $2p_{3/2}$ - 780.9 eV; Co $2p_{1/2}$ - 806.8 eV)[4]. Cu (Cu $2p_{3/2}$ - 933.5 eV; Cu $2p_{1/2}$ - 953.6 eV)[5]. Ir (Ir $4f_{7/2}$ - 61.4 eV; Ir $4f_{5/2}$ - 64.5 eV). Ni (Ni $2p_{3/2}$ - 855.6 eV; Ni $2p_{1/2}$ - 871.9 eV). Pd (Pd $3d_{3/2}$ - 331.3 eV and 341.6 eV; Pd $3d_{5/2}$ - 336.1 eV). Pt (Pt $4f_{5/2}$ - 72 eV; Pt $4f_{7/2}$ - 75.4 eV). Sn (Sn $3d_{5/2}$ - 486.5 eV; Sn $3d_{3/2}$ - 494.7 eV) The phenomenon of the metallic oxidized state should result from the metal on the surface oxidized by air.

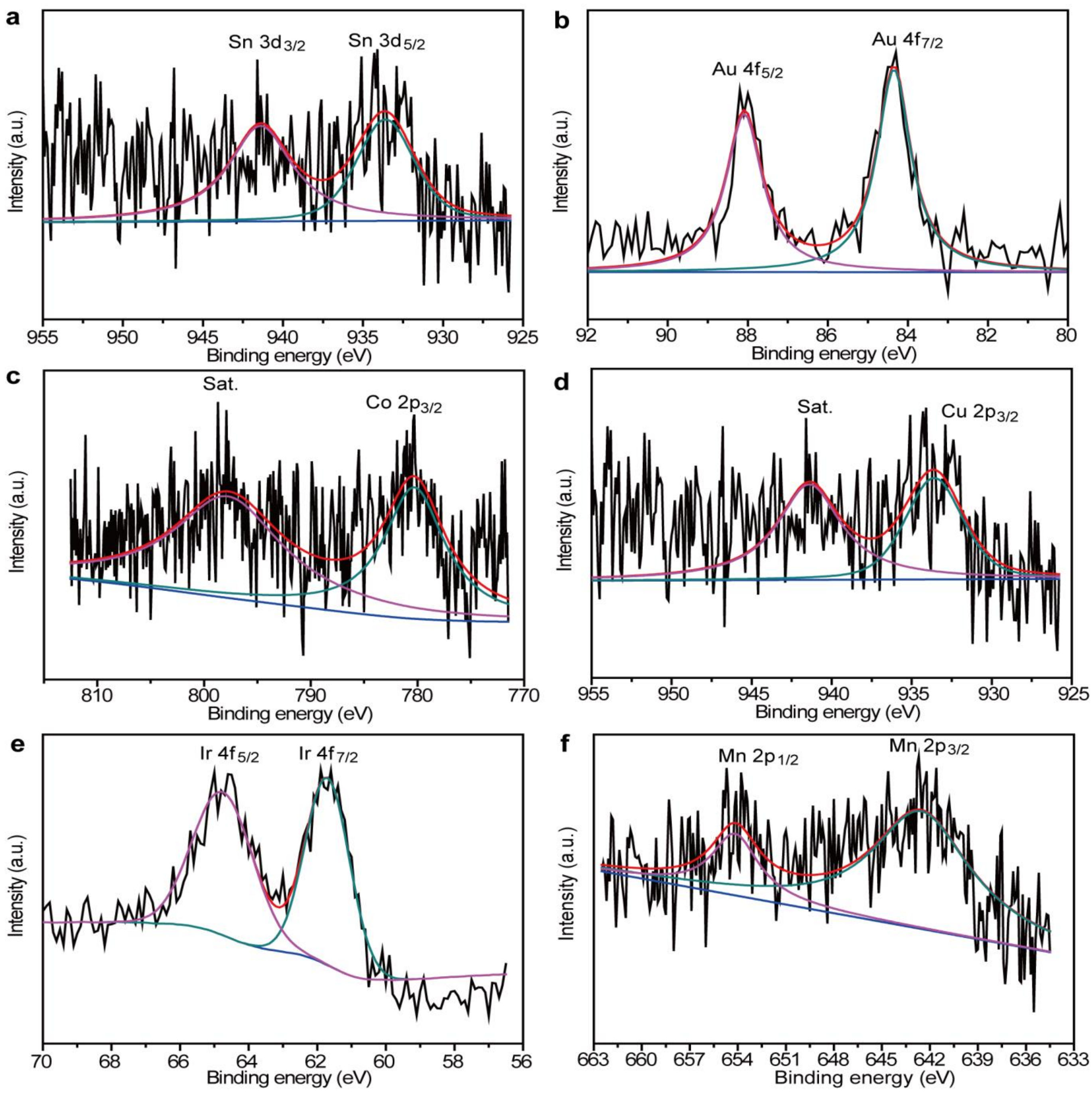

Supplementary Figure 17. XPS spectra for denary (MnCoNiCuRhPdSnIrPtAu) alloy. (**a-f**) The XPS spectra of Sn, Au, Co, Cu, Ir, and Mn for the denary (MnCoNiCuSnRhPdIrPtAu) HEA-NPs. The loading of HEA-NPs on GO was 3 wt%. Sn (Sn $3d_{5/2}$ - 486.6 eV; Sn $3d_{3/2}$ - 495 eV). Au (Au $4f_{7/2}$ - 84.3 eV; Au $4f_{5/2}$ - 88 eV). Co (Co $2p_{3/2}$ - 780.3 eV). Cu (Cu $2p_{3/2}$ - 933.6 eV). Ir (Ir $4f_{7/2}$ - 61.7 eV; Ir $4f_{5/2}$ - 64.8 eV). Mn (Mn $2p_{3/2}$ - 642.6 eV; Mn $2p_{1/2}$ - 654.2 eV).

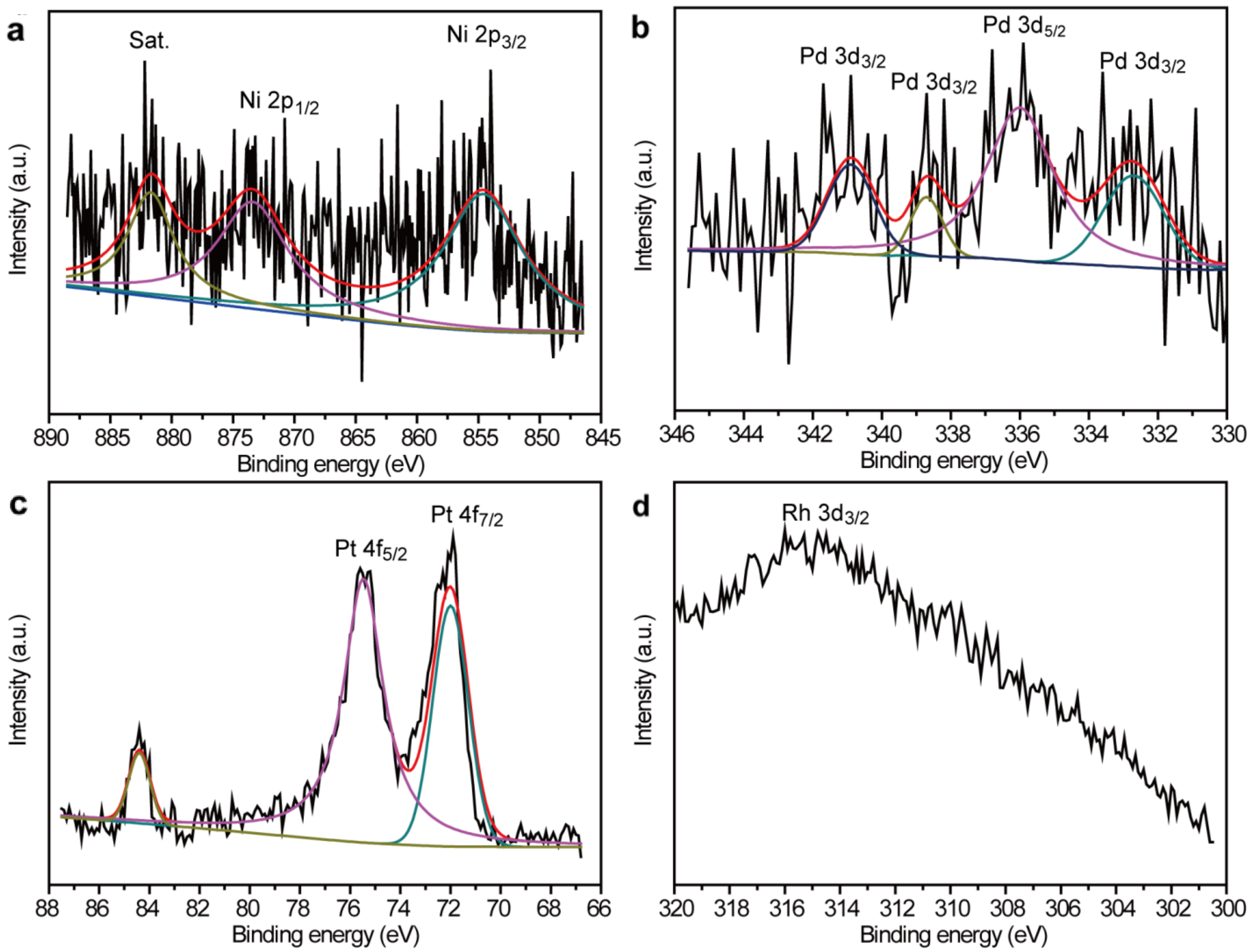

Supplementary Figure 18. XPS spectra for denary (MnCoNiCuRhPdSnIrPtAu) alloy. (**a-d**) The XPS spectra of Ni, Pd, Pt, and Rh for denary (MnCoNiCuSnRhPdIrPtAu) HEA-NPs. The loading of HEA-NPs on GO was 3 wt%. Ni (Ni $2p_{3/2}$ - 854.7 eV; Ni $2p_{1/2}$ - 873.4 eV). Pd (Pd $3d_{3/2}$ - 332.7 eV, 338.7 eV, and 340.9 eV; Pd $3d_{5/2}$ - 336 eV). Pt (Pt $4f_{5/2}$ - 71.9 eV; Pt $4f_{7/2}$ - 75.4 eV). Rh (Rh $3d_{3/2}$ - 314.8 eV).

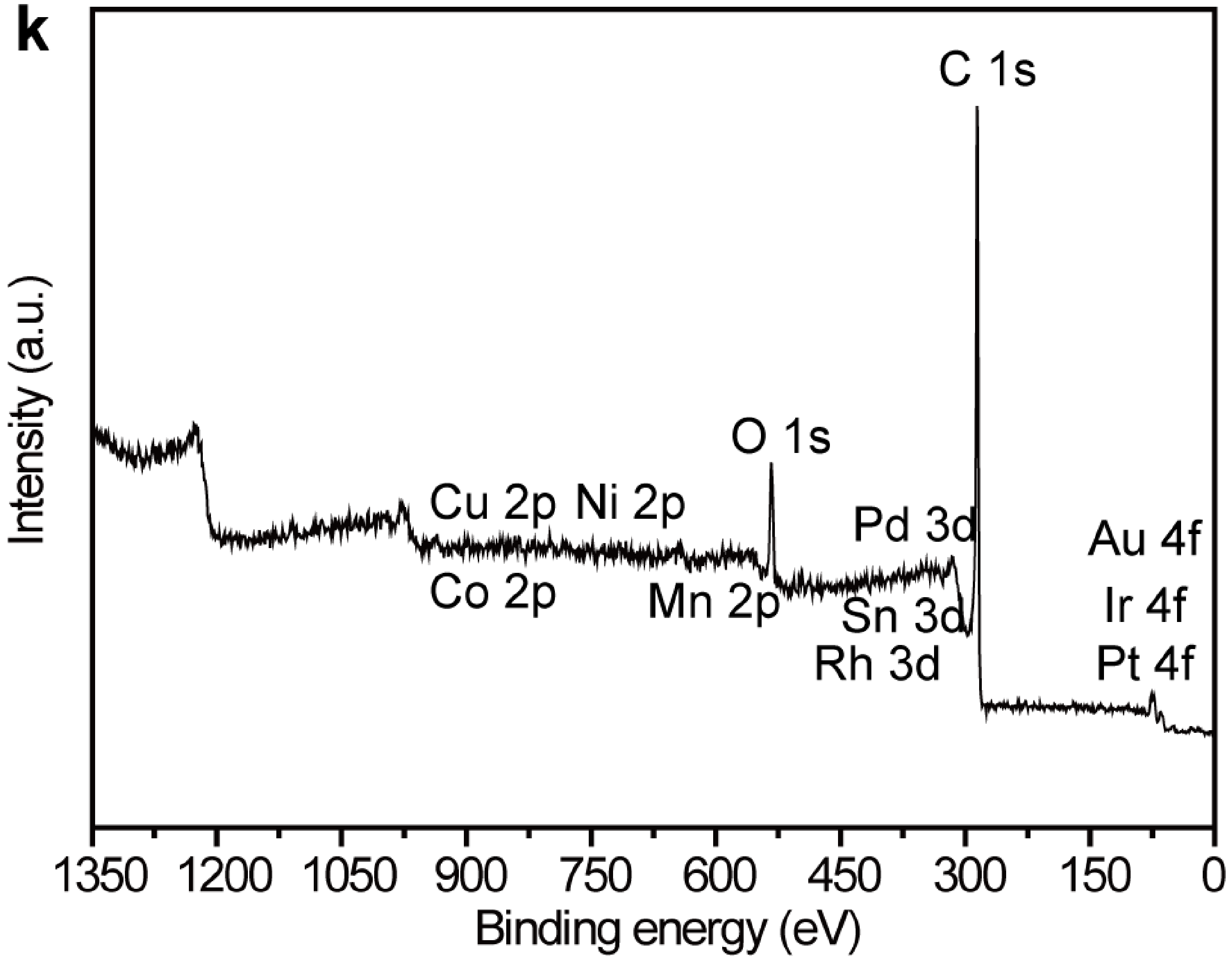

Supplementary Figure 19. XPS spectrum for denary (MnCoNiCuRhPdSnIrPtAu) alloy. The loading of HEA-NPs on GO was 3 wt%.

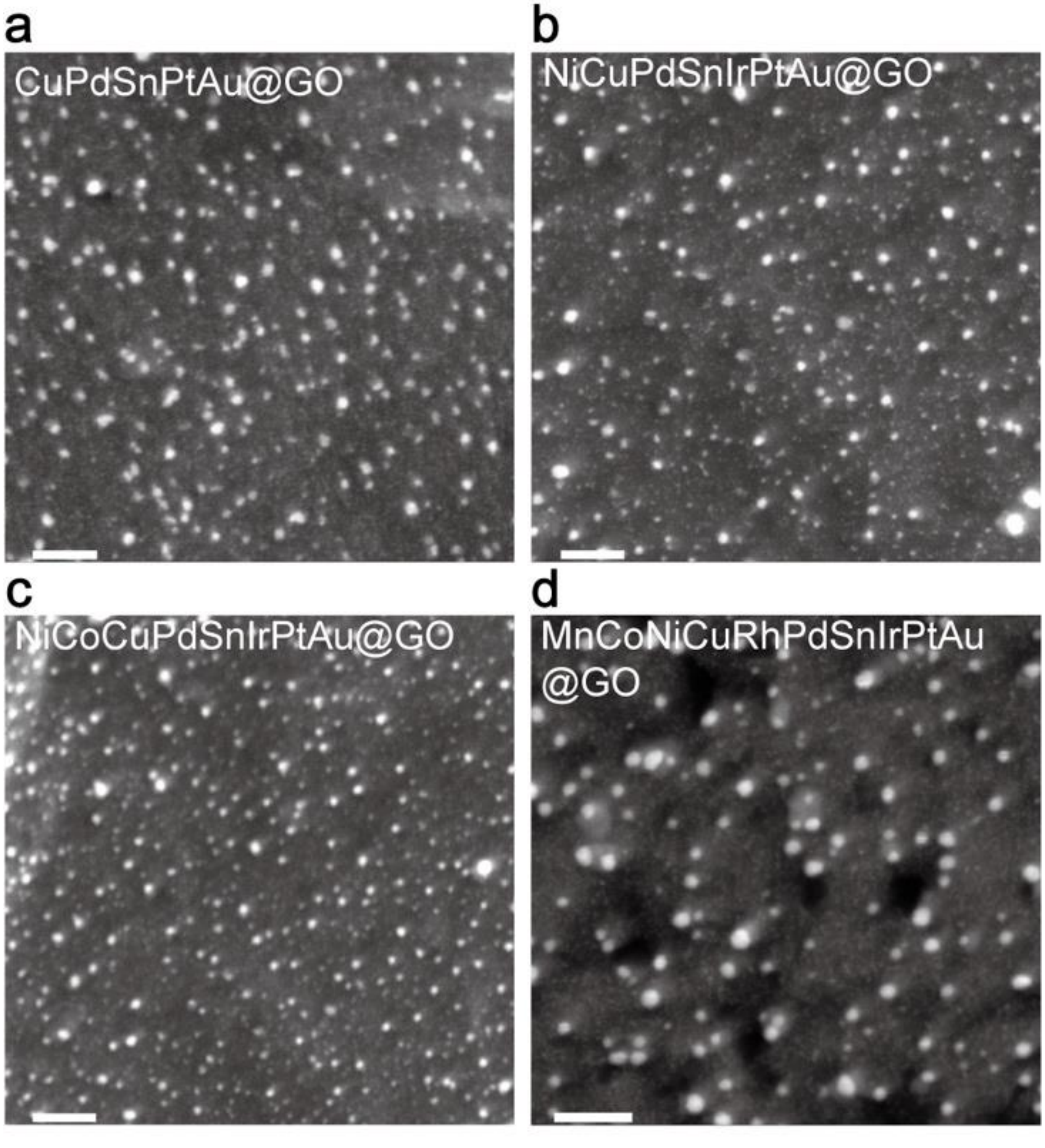

Supplementary Figure 20. STEM images of alloys containing 5-10 metals by FMBP. STEM images of quinary (CuPdSnPtAu) (**a**), septenary (NiCuPdSnIrPtAu) (**b**), octonary (NiCoCuPdSnIrPtAu) (**c**), and denary (MnCoNiCuRhPdSnIrPtAu) (**d**) HEA-NPs supported on GO. The loading of HEA-NPs on GO was 3 wt%. Scale bar (a-d): 10 nm.

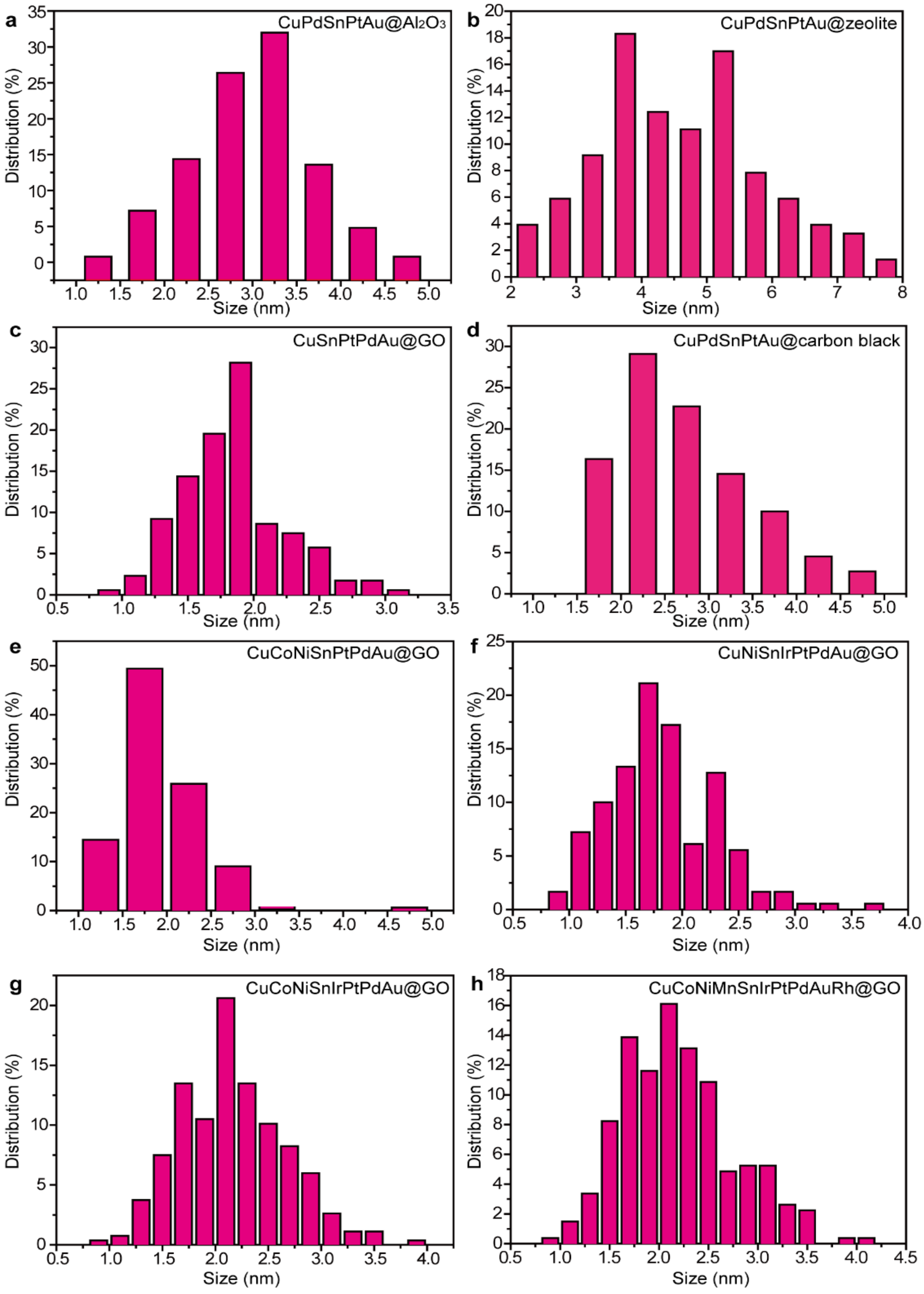

Supplementary Figure 21. Size distribution of HEA-NPs on GO by FMBP at 923K. The loading of HEA-NPs was 3 wt%.

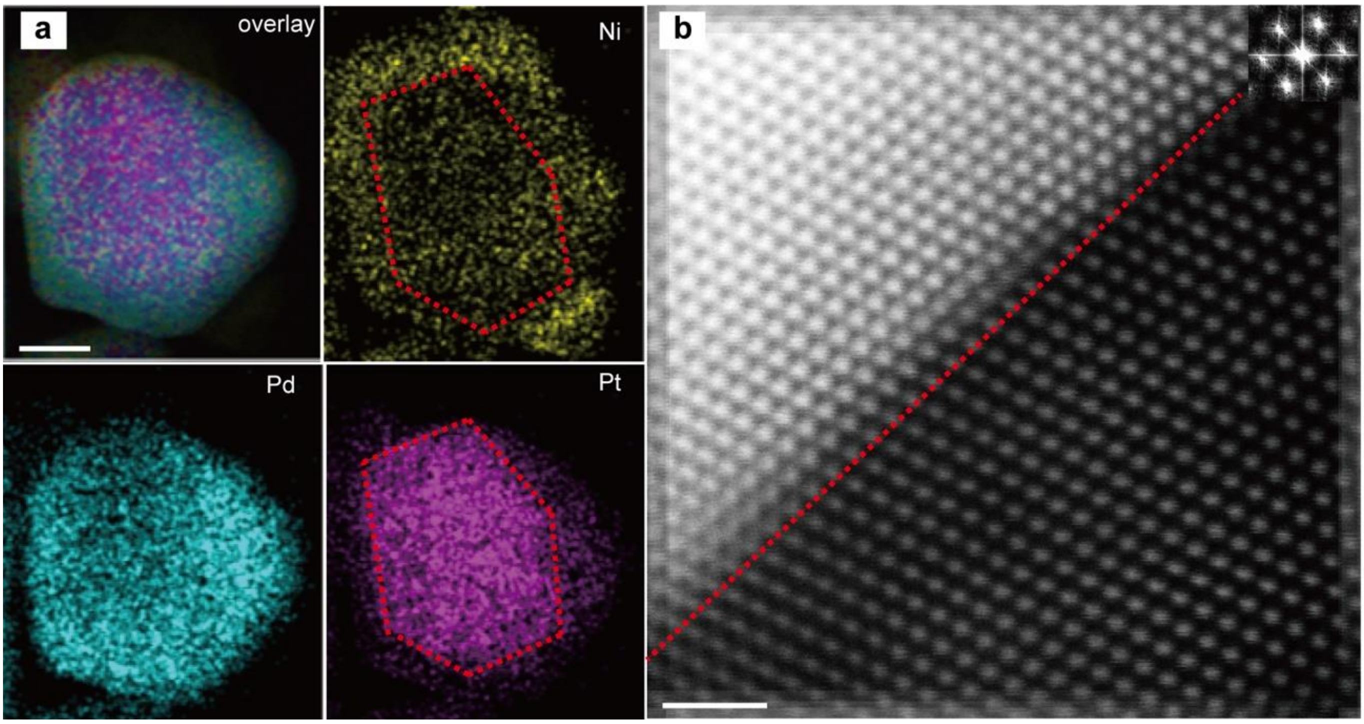

Supplementary Figure 22. The ternary (NiPdPt) alloy synthesized by FBP at 923 K. (**a**) The elemental maps for phase-separated ternary (NiPtPd) alloy. (**b**) The HAADF-STEM image for the phase-separated ternary (NiPtPd) alloy (inset, the Fourier transform analysis for denary HEA-NPs revealed that the ternary alloy featured with an fcc framework). The loading of HEA-NPs on GO was 10 wt%. Scale bar a: 15 nm, b: 1 nm.

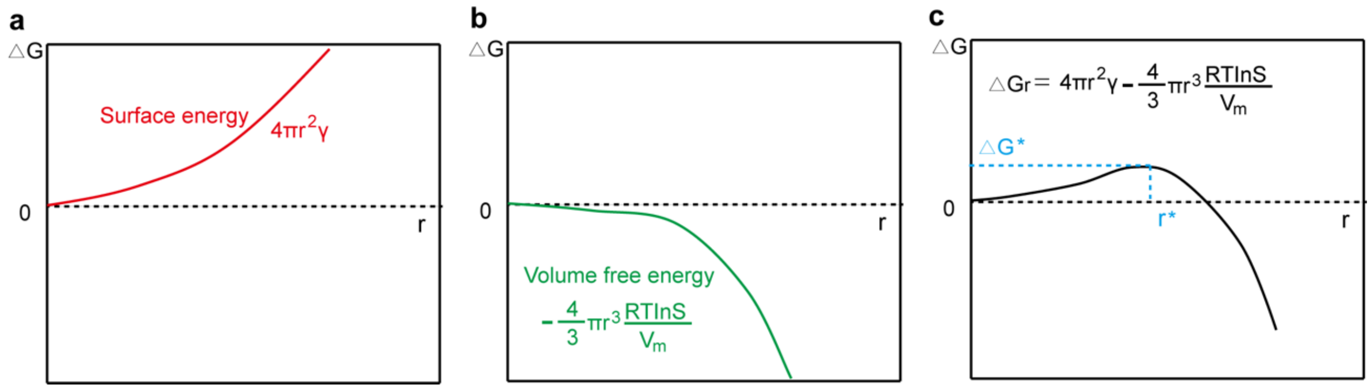

Supplementary Figure 23. Diagram of free energy change versus the nucleus size (r). (**a**) The surface energy vs nucleus size (*r*). (**b**) The volume energy vs *r*. (**c**) Illustration of free energy (ΔG_r) change versus nucleus size *r*.

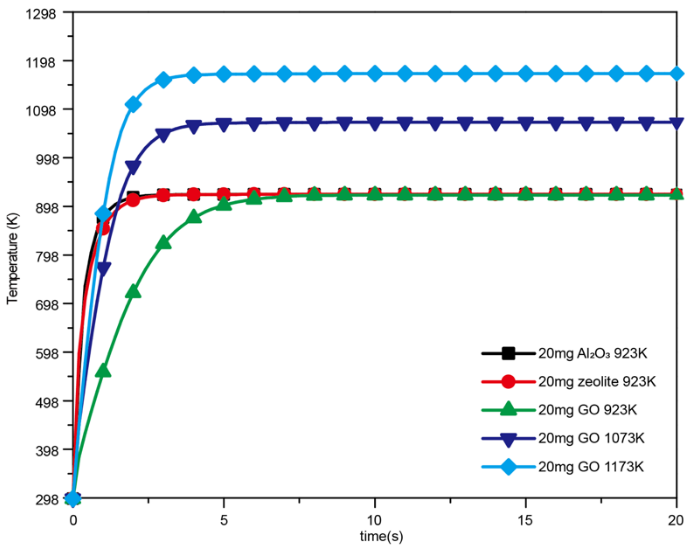

Supplementary Figure 24. Time for various supports reached different temperature. The diagrams of the simulation of the time required for 20 mg of zeolite, γ-Al_2O_3, and GO reaching 923K or 1173 K in the FMBP process.

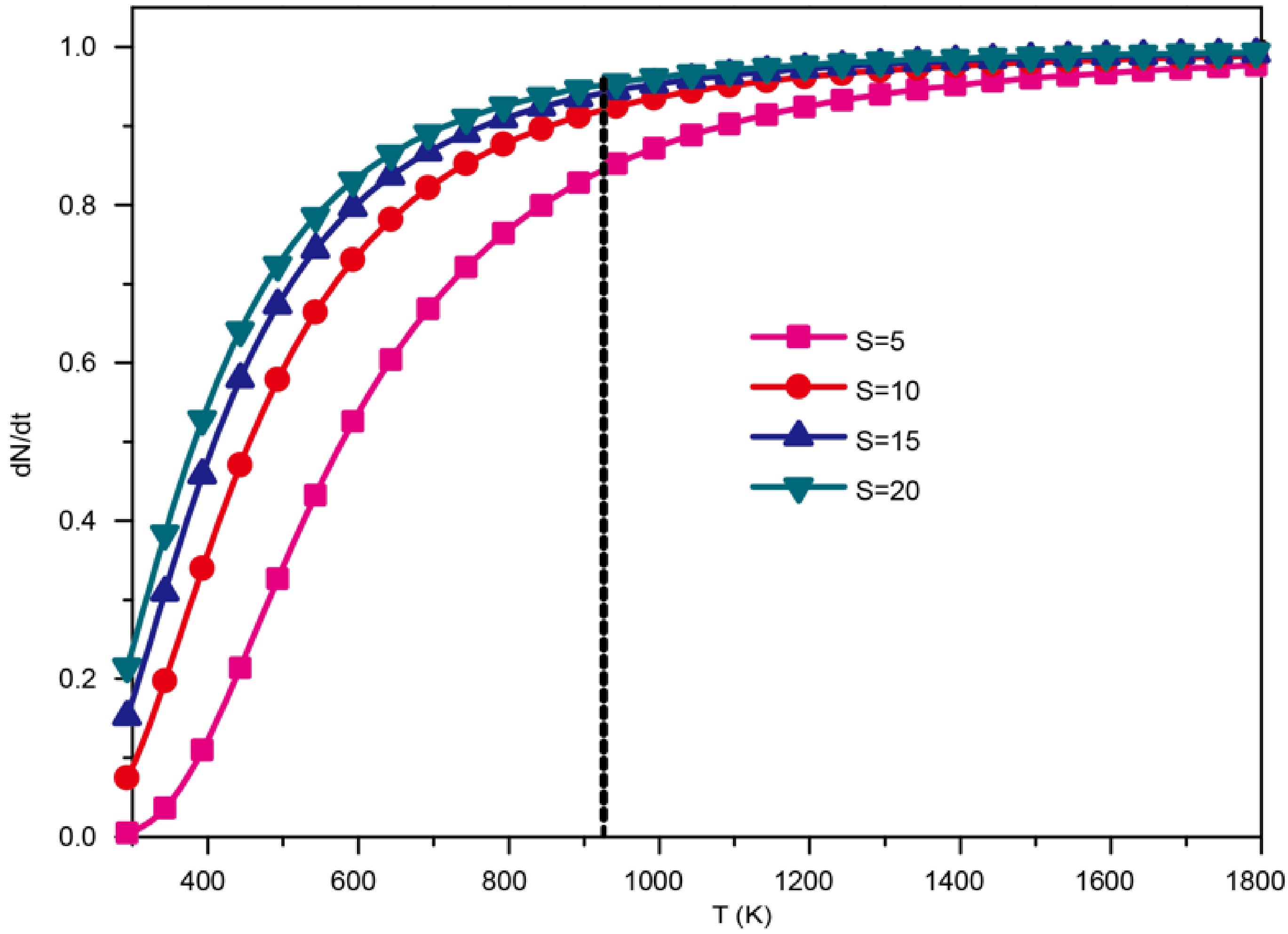

Supplementary Figure 25. Nucleation rate versus temperature. The pre-exponential factor was set as 1.

Supplementary Note 1. The mechanism of nucleation.

As shown in Fig. 5d, the number of the nuclei clusters (N_r) can be calculated from Boltzmann distribution and Equation, the detailed equation can be expressed as[9, 10]:

$$N_r = N_0 \times \exp(-\Delta G_r/RT) = N_A[A]_{eq}S \times \exp(-\Delta G_r/RT) \quad \textbf{(Supplementary Equation 1)}$$

N_0 and N_A respectively represents total amount of free atoms per unit volume of solute and Avogadro constant. A is the pre-exponential factor. S takes for the saturability, $S \geq 1$, when the reactant concentration is oversaturated, $S < 1$, when the reactant concentration is unsaturated.

As illustrated in Fig. 5c, there is a critical excess free energy, ΔG^*, associated with the critical radius of cluster, r^*. When the radius is smaller than the critical value, the system lowers its free energy by dissolving clusters, and at the same time new ones form due to spontaneous collisions [6-8]. The total number of the clusters follows the Boltzmann distribution and decreases rapidly with the increase of cluster radius. When cluster size is greater than the critical radius, system lowers its free energy through continuous growth of clusters. The expressions of critical radius r^*, maximum excess free energy ΔG^*, number of clusters that reaches the critical size Nr^*, and nucleation rate d_{Nr}/d_t can then be written as the followings by solving the equation $d_G/d_r = 0$. Then, Supplementary Equation 1 can be written as follows:

$$\frac{dNr}{dt} = N_A[A]_{eq}Sexp\left[\frac{-16\pi\gamma^3 V_m^2}{3R^3T^3(lnS)^2}\right] = Aexp\left[\frac{-16\pi\gamma^3 V_m^2}{3k_B^3T^3N_A^3(lnS)^2}\right] \quad \textbf{(Supplementary Equation 2)}$$

Where Nr is the number of nuclei, A is the pre-exponential factor, K_B is the Boltzmann constant, N_A is the Avogadro's number, T is temperature, γ is the surface free energy, V_m is the monomer molar volume, S is the level of supersaturation.

In Supplementary Equation 2, there are three experimentally controllable parameters: the level of supersaturation, temperature, and surface free energy.

From Supplementary Equation 2, it can be seen that the effect of these parameters on the nucleation rate. Notably, the nucleation rate is extremely sensitive to the supersaturation level, much more than to the other parameters. The level of supersaturation is increased with temperature (**Supplementary Figure 25**), and the parameters for simulation are shown in Supplementary Tables 4, 5. This strong dependence of the nucleation rate on the supersaturation level plays a very important role.

Supplementary Table 4. The surface free energy for GO, γ-Al_2O_3, and Zeolite.

Substrate	GO	γ-Al_2O_3	Zeolite
Surface free energy γ J m^{-2}	0.0621	=0.2534-0.00036* T (°C)	=0.17342056-0.00015598*T(°C)
S	20	20	20

Supplementary Table 5. The molar volume for these employed metal precursors.

Metal precursors	V_m cm^{-3}
$MnCl_2$	42.37037
$FeCl_3$	55.93241
$CoCl_2$	38.75821
$NiCl_2$	36.50687
$CuCl_2$	50.34849
$RhCl_3$	38.89591
$PdCl_2$	44.3325
$SnCl_4$	117.0261
$IrCl_3$	56.33509
$PtCl_4$	78.29189
$AuCl_3$	77.77564
Average	57.87031

Note: We used the average value of them for calculation.

Supplementary Table 6. The critical radius (r^*) and the critical excess free energy (ΔG_{r^*}) at different temperatures.

Temperature (K)	r^*(nm)	ΔG_{r^*} (J)
923	0.313	2.54×10^{-20}
673	0.442	5.08×10^{-20}

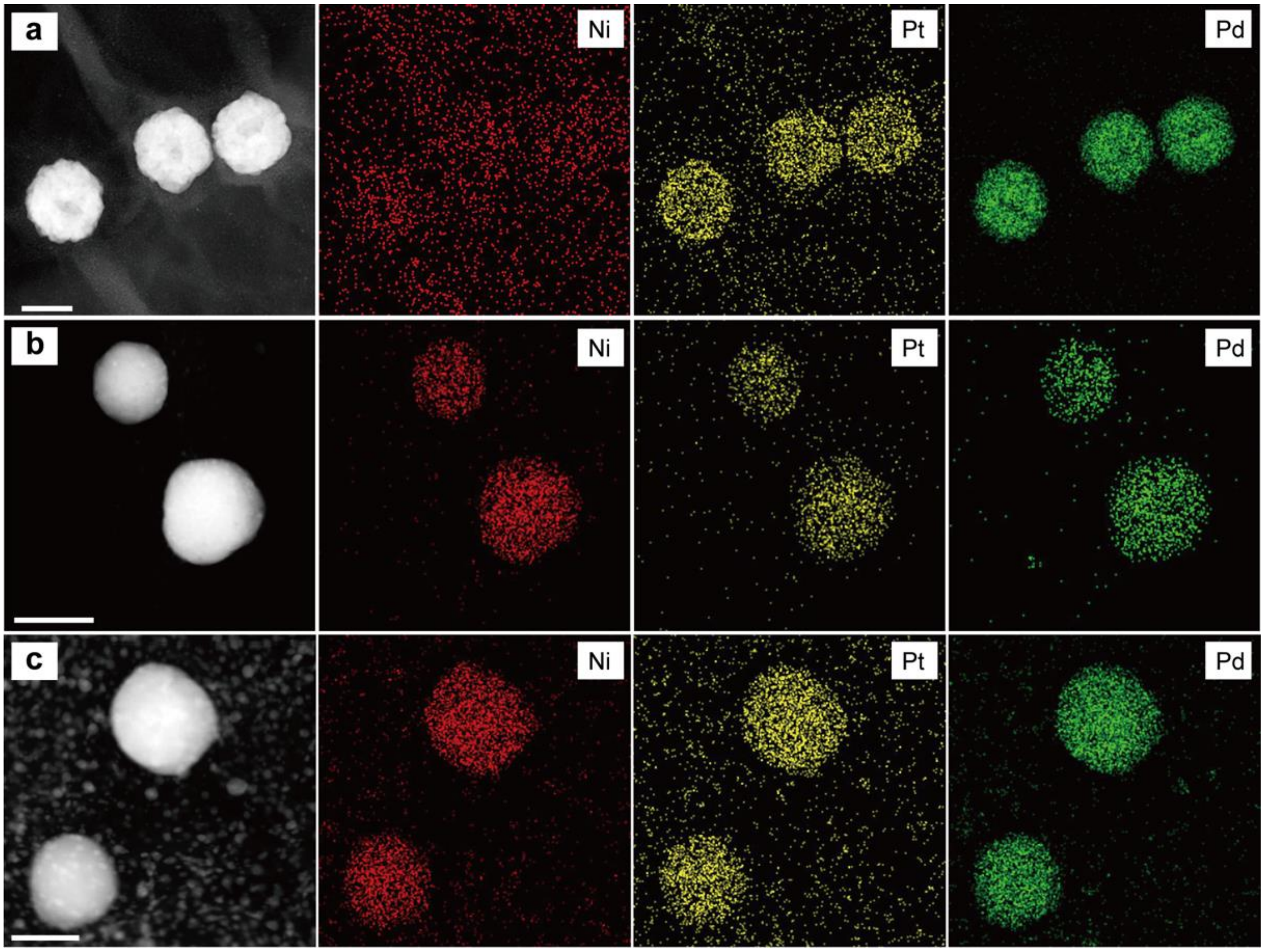

Supplementary Figure 26. Elemental maps of NiPdPt by FMBP at different temperature. The ternary (NiPdPt) alloy supported on GO synthesized by the FMBP method at 673 K (**a**), 923 K (**b**), and 1173 K (**c**), respectively. The loading of HEA-NPs on GO was 10 wt%. Scale bar (a-b): 50 nm, c: 70 nm.

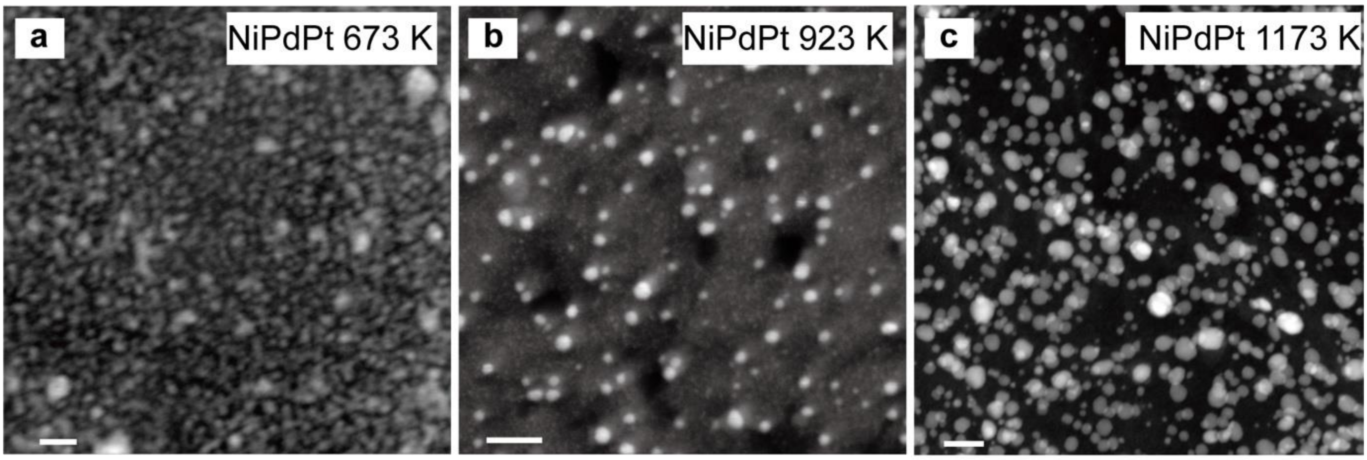

Supplementary Figure 27. STEM images for NiPdPt by FMBP at different temperature. The ternary (NiPdPt) alloy supported on GO synthesized by the FMBP (120 min) method at 673 K (**a**), 923 K (**b**), and 1173 K (**c**), respectively. The loading of HEA-NPs on GO was 3 wt%. Scale bar (a-b): 10 nm, c: 20 nm.

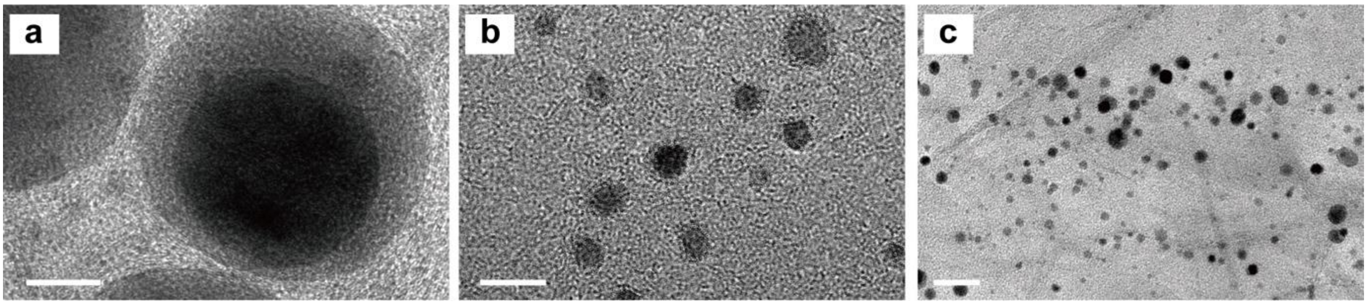

Supplementary Figure 28. STEM images for CuSnPdPtAu by FMBP for different time. (**a**) 30 min, (**b**) 120 min, (**c**) 180 min. The loading of HEA-NPs on GO was 3 wt%. Scale bar (a-b): 10 nm, c: 20 nm.

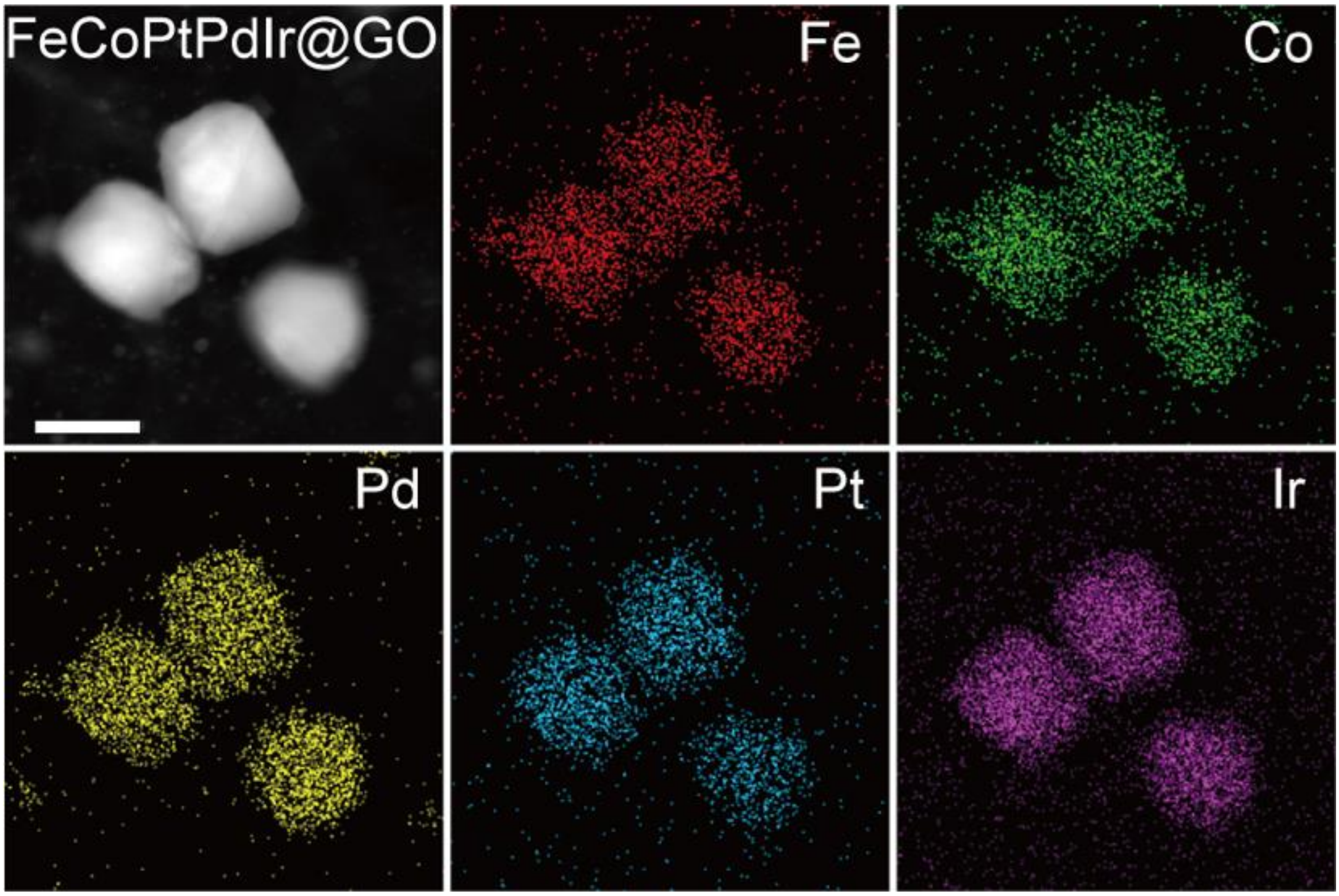

Supplementary Figure 29. Elemental maps for FeCoPdIrPt supported on GO. The loading of HEA-NPs on GO was 10 wt%. Scale bar: 50 nm.

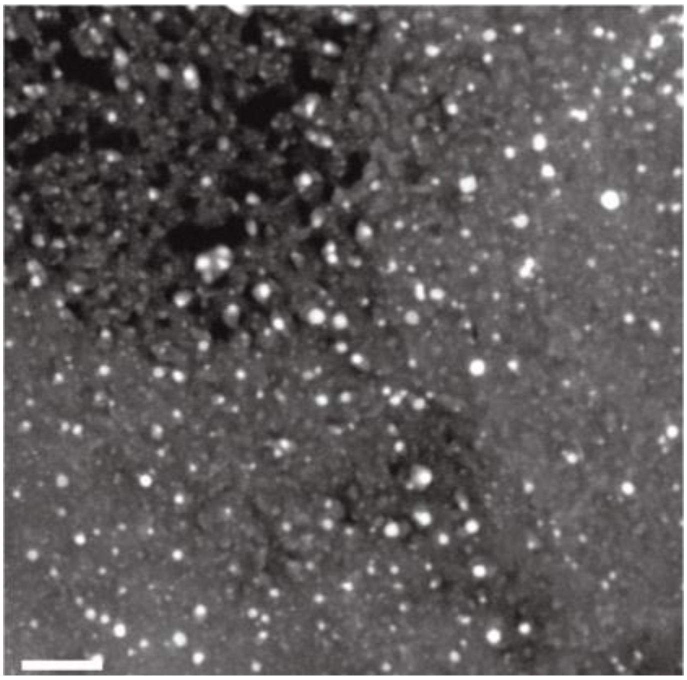

Supplementary Figure 30. STEM image for FeCoPdIrPt supported on GO. The loading of HEA-NPs on GO was 3 wt%. Scale bar: 20 nm.

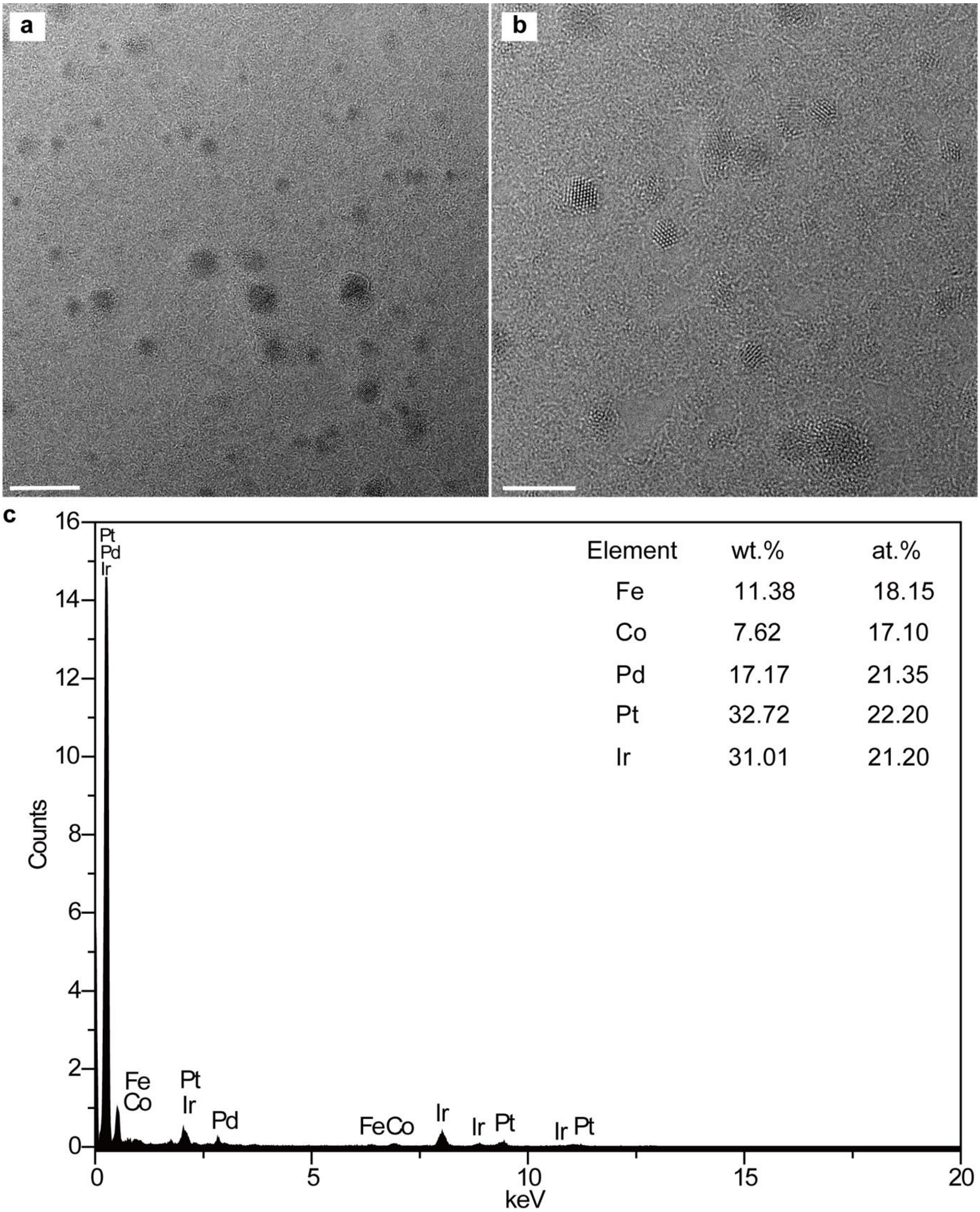

Supplementary Figure 31. HRTEM images and EDX spectra for FeCoPdIrPt on GO. HRTEM images (**a**, **b**) and EDX spectrum (**c**) for the quinary (FeCoPdPtIr) HEA-NPs supported on GO by the FMBP at 923 K. The loading of HEA-NPs on GO was 3 wt%. Scale bar a: 10 nm, b: 5 nm.

Supplementary Table 7. Chemical reduction potentials and reductive pyrolysis temperatures of the used precursor salts in this work.

Precursors	Chemical reduction potentials (V) [11-18]	Reductive pyrolysis temperatures (K) [19-26]
$MnCl_2$	-1.18	873
$CoCl_2$	-0.28	673
$NiCl_2$	-0.26	673
$CuCl_2$	0.34	673
$SnCl_4$	-0.14	600
$RhCl_3$	0.76	673
$PdCl_2$	0.95	573
$IrCl_3$	1.12	673
H_2PtCl_6	1.18	450
$HAuCl_4$	1.5	773

The reductive pyrolysis temperatures of these metal salts were referred to the temperatures employed for preparation their pure metal or alloy.

Supplementary Table 8. Physical properties of metals used in this study.

Element	Pauling electronegativity[27, 28]	Atomic Radius (Å)[29]
Mn	1.55	1.61
Fe	1.83	1.56
Co	1.88	1.52
Ni	1.91	1.49
Cu	1.90	1.45
Rh	2.28	1.73
Sn	1.96	1.45
Pd	2.20	1.69
Ir	2.20	1.80
Pt	2.28	1.77
Au	2.54	1.74

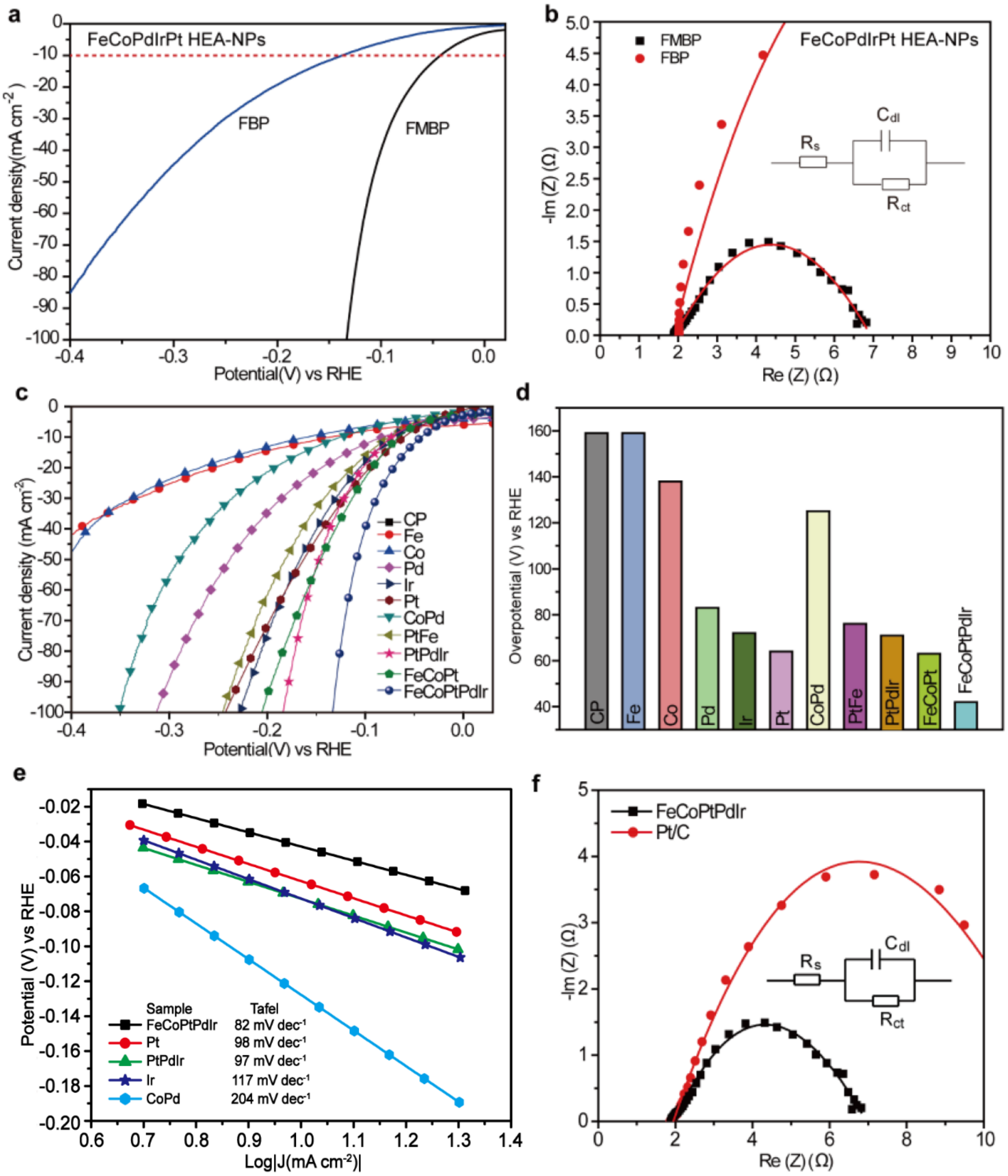

Supplementary Figure 32. Electrochemical performance comparison of samples. (**a**) Linear sweep voltammetry (LSV) curves of FeCoPtPdIr@GO prepared by FMBP and FBP. (**b**) EIS plot for the quinary FeCoPtPdIr@GO prepared by FMBP and FBP. (**c**) LSV curves of the single-metallic, bi-metallic, tri-metallic, and quinary samples supported on GO. (**d**), The comparison overpotential of the samples at 10 mA cm^{-2}. (**e**) The Tafel plots for samples. (**f**) The EIS plot for the quinary (FeCoPtPdIr) HEA-NPs and the commercial Pt/C.

Supplementary Figure Table 9. Comparison of representative electrocatalysts by different methods for HER performance in alkaline solution.

Sample	Over Potential η for 10 $mA \cdot cm^{-2}$ (mV)	Mass activity $(mA\ \mu g_{metal}^{-1})$	Stability	Methods	Reference
FeCoPdIrPt NPs	42	9.1(Pt)<br>3.6(PtPdIr)<br>$(\eta = 100\ mV)$	100 $mA \cdot cm^{-2}$<br>150 h	FMBP	This study
Pd-Pt-T with Pd (111)-Pt interface	94	0.525<br>$(\eta = 100 mV)$	70 mV<br>8000 s	Electrospinning	30
β-$Ni(OH)_2$/Pt	92	0.615<br>$(\eta = 100\ mV)$	90 mV<br>3.5h	Hydrothermal	31
Pd-CN_x	150	0.186<br>$(\eta = 100\ mV)$	10 $mA \cdot cm^{-2}$<br>45 h	$NaBH_4$ reduction	32
Pd/$FeO_x(OH)_{2-2x}$	150	0.08<br>$(\eta = 150\ mV)$	100 mV<br>2 h	Solvothermal	33
NiMoN	109	0.01<br>$(\eta = 100 mV)$	30 $mA \cdot cm^{-2}$<br>36 h	RF Plasma	34
Sr_2RuO_4	61	0.375<br>$(\eta = 100\ mV)$	10 $mA \cdot cm^{-2}$<br>10 h	Solid state reaction	35
Pt/$Ni(HCO_3)_2$	106	1.77<br>$(\eta = 100 mV)$	100 mV<br>11000s	Hydrothermal	36
Ni-N-C	147	0.1<br>$(\eta = 147\ mV)$	300 mV<br>10 h	Corrosion	37
NiCoN/C	103	0.204<br>$(\eta = 200 mV)$	200 mV<br>10h	Corrosion	38
Pt_2Pd/nitrogen-rich graphene nanopores	58	0.94<br>$(\eta = 80 mV)$	150 $mA \cdot cm^{-2}$<br>10000s	Hydrothermal	39
AuPt@Pt	116	1.85<br>$(\eta = 200\ mV)$	70 $mA \cdot cm^{-2}$<br>2 h	Slow reduction	40
Co-BDC/MoS_2	248	0.224<br>$(\eta = 100\ mV)$	10 $mA \cdot cm^{-2}$<br>5 h	Sonication	41

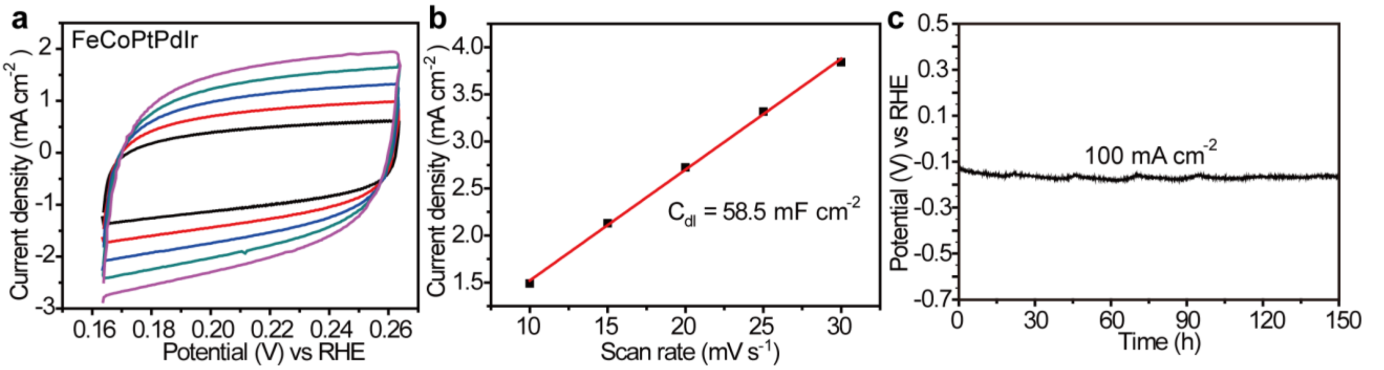

Supplementary Figure 33. CV curves for the quinary (FeCoPdIrPt) HEA-NPs. (**a**) CV curves for the quinary (FeCoPtPdIr) HEA-NPs, scan rate of 10-30 mV·s^{-1}. (**b**) C_{dl} measurements for the quinary (FeCoPtPdIr) HEA-NPs in HER. (**c**) Chronopotentiometry of FeCoPdPtIr@GO at constant 100 mA·cm^{-2}.

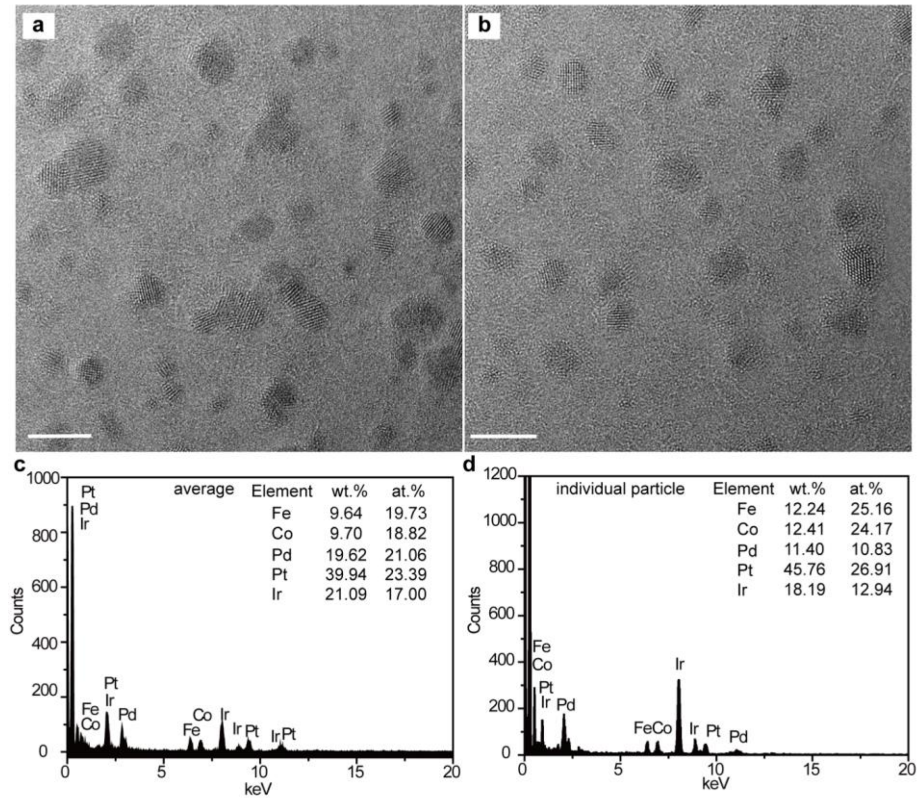

Supplementary Figure 34. HRTEM images and EDX spectra for FeCoPdIrPt after HER test. (**a, b**) HRTEM images for the quinary (FeCoPdPtIr) HEA-NPs. (**c**) EDX spectra for the quinary (FeCoPdPtIr) HEA-NPs supported on GO after HER. (**d**) EDX spectra for the individual FeCoPdPtIr HEA-NP supported on GO after HER reaction after HER stability test. Scale bar a: 10 nm, b: 5 nm.

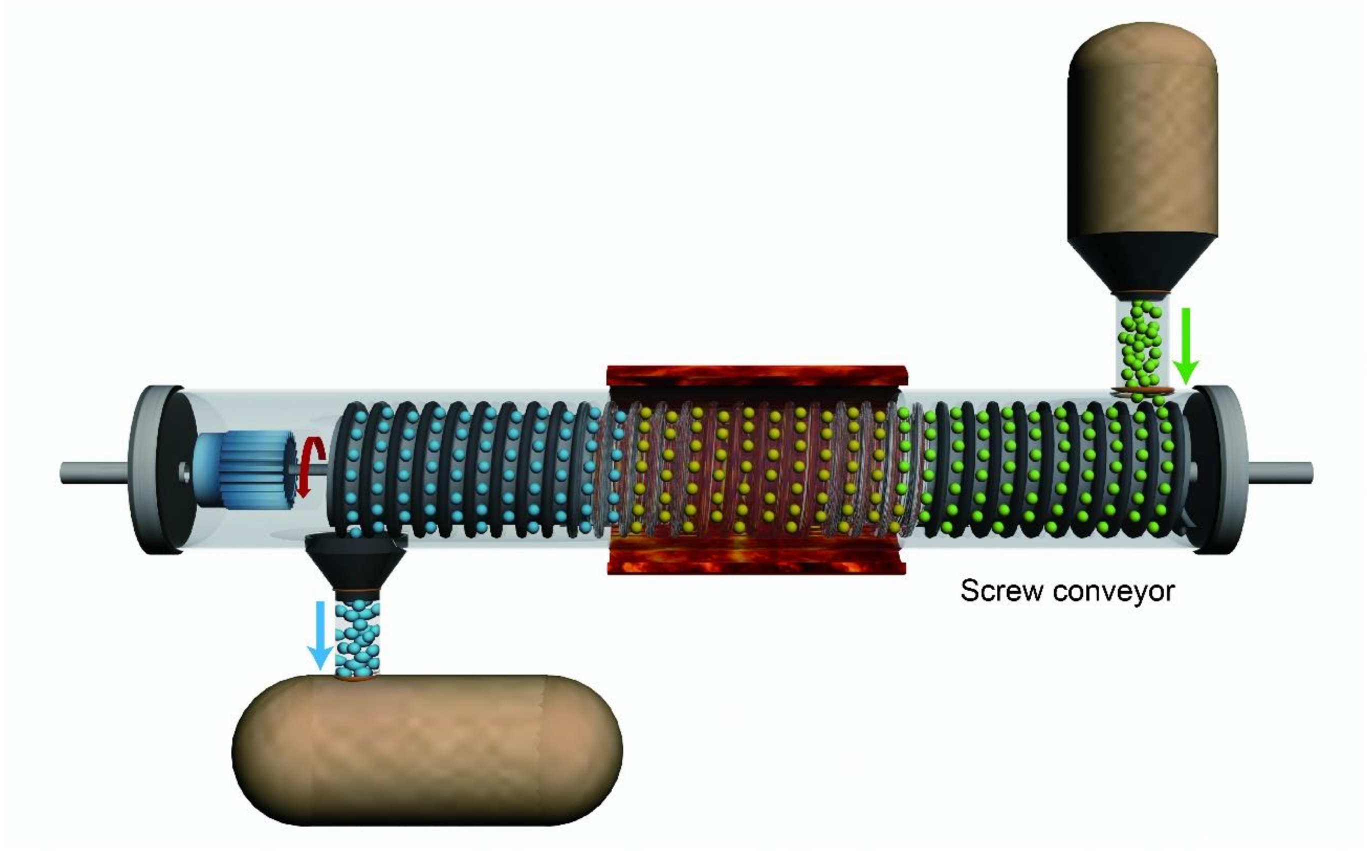

Supplementary Figure 35. Scalable production of supported HEA-NPs by FMBP.

Supplementary References:

1. de Pedro, Z. M., Diaz, E., Mohedano, A. F., Casas, J. A. & Rodriguez, J. J. Compared activity and stability of Pd/Al_2O_3 and Pd/AC catalysts in 4-chlorophenol hydrodechlorination in different pH media. *Appl. Catal. B-Environ.* **103**, 128-135 (2011).

2. Bera, P. et al. Promoting effect of CeO_2 in combustion synthesized Pt/CeO_2 catalyst for CO oxidation. *The J. Phys. Chem. B* **107**, 6122-6130 (2003).

3. Brown, M. A. et al. Oxidation of Au by surface OH: nucleation and electronic structure of gold on hydroxylated MgO(001). *J. Am. Chem. Soc.* **133**, 10668-10676 (2011).

4. Andreiadis, E. S. et al. Molecular engineering of a cobalt-based electrocatalytic nanomaterial for H_2 evolution under fully aqueous conditions. *Nat. Chem.* **5**, 48-53 (2012).

5. Kim, J. et al. Branched copper oxide nanoparticles induce highly selective ethylene production by electrochemical carbon dioxide reduction. *J. Am. Chem. Soc.* **141**, 6986-6994 (2019).

6. LaMer, V. K. & Dinegar, R. H. Theory, production and mechanism of formation of monodispersed hydrosols. *J. Am. Chem. Soc.* **72**, 4847-4854 (1950).

7. Bøjesen, E. D. & Iversen, B. B. The chemistry of nucleation. *CrystEngComm* **18**, 8332-8353 (2016).

8. Watzky, M. A. & Finke, R. G. Transition metal nanocluster formation kinetic and mechanistic studies. a new mechanism when hydrogen is the reductant: slow, continuous nucleation and fast autocatalytic surface growth. *J. Am. Chem. Soc.* **119**, 10382-10400 (1997).

9. Shevchenko, E. V. et al. Study of nucleation and growth in the organometallic synthesis of magnetic alloy nanocrystals: the role of nucleation rate in size control of $CoPt_3$ nanocrystals. *J. Am. Chem. Soc.* **125**, 9090-9101 (2003).

10. Peng, Z. & Yang, H. Designer platinum nanoparticles: control of shape, composition in alloy, nanostructure and electrocatalytic property. *Nano Today* **4**, 143-164 (2009).

11. Kozlov Yu, N., Kazakova, A. A. & Klimov, V. V. Changes in the redox potential and catalase activity of

Mn^{2+} ions during formation of Mn-bicarbonate complexes. *Membr. Cell bio.* **11**, 115-120 (1997).

12. Üzüm, Ç. et al. Synthesis and characterization of kaolinite-supported zero-valent iron nanoparticles and their application for the removal of aqueous Cu^{2+} and Co^{2+} ions. *Appl. Clay Sci.* **43**, 172-181 (2009).
13. Mani, P., Srivastava, R. & Strasser, P. Dealloyed binary PtM_3 (M=Cu, Co, Ni) and ternary $PtNi_3M$ (M=Cu, Co, Fe, Cr) electrocatalysts for the oxygen reduction reaction: performance in polymer electrolyte membrane fuel cells. *J. Power Sources* **196**, 666-673 (2011).
14. Latimer, W. M. The oxidation states of the elements and their potentials in aqueous solutions. **48**, LWW (1939).
15. Zhang, Z. P., Zhu, W., Yan, C. H. & Zhang, Y.-W. Selective synthesis of rhodium-based nanoframe catalysts by chemical etching of 3d metals. *Chem. Commun.* **51**, 3997-4000 (2015).
16. Xu, L. et al. Replacement reaction-based synthesis of supported palladium catalysts with atomic dispersion for catalytic removal of benzene. *J. Mater. Chem. A* **6**, 17032-17039 (2018).
17. Hwang, S. J. et al. Facile synthesis of highly active and stable Pt–Ir/C electrocatalysts for oxygen reduction and liquid fuel oxidation reaction. *Chem. Commun.* **46**, 8401-8403 (2010).
18. Song, H. M., Moosa, B. A. & Khashab, N. M. Water-dispersable hybrid Au–Pd nanoparticles as catalysts in ethanol oxidation, aqueous phase Suzuki–Miyaura and Heck reactions. *J. Mater. Chem.* **22**, 15953-15959 (2012).
19. Petrik, I. S. et al. XPS and TPR study of sol-gel derived M/TiO_2 powders (M=Co, Cu, Mn, Ni). *Chemistry, Physics & Technology of Surface / Khimiya, Fizyka ta Tekhnologiya Poverhni* **6**, 179-189 (2015).
20. Xu, G., Zhu, Y., Ma, J., Yan, H. & Xie, Y. TPR of Pd/MnO_2 and Pd/Fe_2O_3 systems-effects of hydrogen spillover. In: Li C, Xin Q (eds). *Stud. Surf. Sci. Catal.* **112**, 333-338 (1997).
21. Campos, C. et al. Hydrogenation of substituted aromatic nitrobenzenes over 1% 1.0wt.%Ir/ZrO_2 catalyst: Effect of meta position and catalytic performance. *Catal. Today* **213**, 93-100 (2013).

22. Shirai, M., Igeta, K. & Arai, M. The preparation and structure of platinum metal nanosheets between graphite layers. *J. Phys. Chem. B* **105**, 7211-7215 (2001).

23. Xu, X. L., Xu, X. F., Zhang, G. T. & Niu, X. J. Preparation of Co-Al mixed oxide-supported gold catalysts and their catalytic activity for N_2O decomposition. *J. Fuel Chem. Technol.* **37**, 595-600 (2009).

24. Wang, S. et al. Pd-Fe/α-Al_2O_3/cordierite monolithic catalysts for the synthesis of dimethyl oxalate: effects of calcination and structure. *Front. Chem. Sci. Eng.* **6**, 259-269 (2012).

25. Liu, M. et al. Entropy-maximized synthesis of multimetallic nanoparticle catalysts via a ultrasonication-assisted wet chemistry method under ambient conditions. *Adv. Mater. Interf.* **6**, 1900015 (2019).

26. Wong, A., Liu, Q., Griffin, S., Nicholls, A. & Regalbuto, J. R. Synthesis of ultrasmall, homogeneously alloyed, bimetallic nanoparticles on silica supports. *Science*, **358**, 1427 (2017).

27. Salishchev G.A. et al. Effect of Mn and V on structure and mechanical properties of high-entropy alloys based on CoCrFeNi system. *J Alloy. Compd.* **591**, 11-21 (2014).

28. Jayaprakash R. & Shanker J. Correlation between electronegativity and high temperature superconductivity. *J. Phys. Chem. Solid.* **54**, 365-369 (1993).

29. Slater J.C. Atomic Radii in Crystals. *J. Chem. Phys.* **41**, 3199-3204 (1964).

30. Fan, J. et al. Engineering Pt/Pd interfacial electronic structures for highly efficient hydrogen evolution and alcohol oxidation. *ACS Appl. Mater. Interfaces* **9**, 18008-18014 (2017).

31. Yu, X. et al. Hydrogen evolution reaction in alkaline media: alpha- or beta-nickel hydroxide on the surface of platinum? *ACS Energy Lett.* **3**, 237-244 (2018).

32. Bhowmik, T., Kundu, M. K. & Barman, S. Palladium nanoparticle–graphitic carbon nitride porous synergistic catalyst for hydrogen evolution/oxidation reactions over a broad range of pH and correlation of its catalytic activity with measured hydrogen binding energy. *ACS Catal.* **6**, 1929-1941 (2016).

33. Liao, H. et al. A multisite strategy for enhancing the hydrogen evolution reaction on a nano-Pd Surface in

alkaline media. *Adv. Energy Mater.* **7**, 1701129 (2017).

34. Zhang, Y. et al. 3D porous hierarchical nickel–molybdenum nitrides synthesized by RF plasma as highly active and stable hydrogen-evolution-reaction electrocatalysts. *Adv. Energy Mater.* **6**, 1600221 (2016).

35. Zhu, Y. et al. Unusual synergistic effect in layered ruddlesden−popper oxide enables ultrafast hydrogen evolution. *Nat. Commun.* **10**, 149 (2019).

36. Lao, M. et al. Platinum/nickel bicarbonate heterostructures towards accelerated hydrogen evolution under alkaline conditions. *Angew. Chem. Int. Ed.* **58**, 5432-5437 (2019).

37. Lei, C. et al. Efficient alkaline hydrogen evolution on atomically dispersed Ni–Nx species anchored porous carbon with embedded Ni nanoparticles by accelerating water dissociation kinetics. *Energy Environ. Sci.* **12**, 149-156 (2019).

38. Lai, J., Huang, B., Chao, Y., Chen, X. & Guo, S. Strongly coupled nickel–cobalt nitrides/carbon hybrid nanocages with Pt-Like activity for hydrogen evolution catalysis. *Adv. Mater.* **31**, 1805541 (2019).

39. Zhong, X. et al. PtPd alloy embedded in nitrogen-rich graphene nanopores: High-performance bifunctional electrocatalysts for hydrogen evolution and oxygen reduction. *Carbon* **114,** 740-748 (2017).

40. Shao, F.-Q. et al. Simple fabrication of core-shell AuPt@Pt nanocrystals supported on reduced graphene oxide for ethylene glycol oxidation and hydrogen evolution reactions. *Electrochim. Acta* **219**, 321-329 (2016).

41. Zhu, D., Liu, J., Zhao, Y., Zheng, Y. & Qiao, S.-Z. Engineering 2D metal–organic framework/MoS_2 interface for enhanced alkaline hydrogen evolution. *Small* **15**, 1805511 (2019).
