# Supplementary material for: Synthesis of high-entropy alloy nanoparticles on supports by the fast moving bed pyrolysis
Source: Nat Commun. 2020 Apr 24;11:2016. doi: 10.1038/s41467-020-15934-1 (PMC7181682; doi:10.1038/s41467-020-15934-1)
Supplement: Supplementary file 2 — Description of Additional Supplementary Files [file 41467_2020_15934_MOESM2_ESM.pdf]

Description of Additional Supplementary Files

1. File Name: Supplementary Movie 1

   Description: This movie shows the temperature changes in the furnace by simulation with the FLUENT software.

2. File Name: Supplementary Movie 2

   Description: This movie shows the temperature changes in the furnace under actual operation during synthesis.
